# Effect of Nitrogen and Phosphorus Doping of Reduced Graphene Oxide in the Hydrogen Evolution Catalytic Activity of Supported Ru Nanoparticles

**DOI:** 10.1021/acsami.4c15547

**Published:** 2025-01-20

**Authors:** Laura Mallón, Javier Navarro-Ruiz, Christian Cerezo-Navarrete, Nuria Romero, Iker del Rosal, Jordi García-Antón, Roger Bofill, Luis M. Martínez-Prieto, Karine Philippot, Romuald Poteau, Xavier Sala

**Affiliations:** † Departament de Química, 16719 Universitat Autònoma de Barcelona, 08193 Bellaterra, Catalonia, Spain; ‡ Université de Toulouse; INSA, UPS, CNRS; LPCNO (IRSAMC), 135 avenue de Rangueil, F-31077 Toulouse, France; § ITQ, Instituto de Tecnología Química (CSIC-Universitat Politècnica de València), Av. de los Naranjos S/N, 46022 Valencia, Spain; ∥ CNRS, LCC (Laboratoire de Chimie de Coordination), UPR8241, Université de Toulouse, UPS, INPT, Toulouse cedex 4 F-31077, France; ⊥ IIQ, Instituto de Investigaciones Químicas (CSIC-Universidad de Sevilla), Avda. Américo Vespucio 49, 41092 Seville, Spain

**Keywords:** reduced graphene oxide, N-doping, P-doping, hydrogen evolution reaction, Ru nanoparticles, DFT simulations

## Abstract

Three different cathodic materials for the hydrogen evolution reaction (HER) consisting of Ru nanoparticles (NPs) supported onto a bare and two doped reduced graphene oxides (r-GO) have been studied. Ru NPs have been synthesized in situ by means of the organometallic approach in the presence of each reduced graphene support (bare (rGO), N-doped (NH_2_-rGO) and P-doped (P-rGO)). (HR)­TEM, EDX, EA, ICP-OES, XPS, Raman and NMR techniques have been used to fully characterize the obtained rGO-supported Ru materials. These materials have been deposited onto a glassy carbon rotating disk electrode (GC-RDE) to assess their HER electrocatalytic activity at acidic pH. The results show that all three materials are stable under reductive conditions for at least 12 h, and that the heteroatom-doping of the graphene structure extremely increases the activity of the electrodes, especially for the case of Ru@P-rGO, where the overpotential at −10 mA·cm^–2^ decreases to only 2 mV. *Realistic* (based on experimental compositional data) modeling of the three rGO supports combined with DFT computational analysis of the electronic and electrocatalytic properties of the hybrid nanocatalysts allows attributing the observed electrocatalytic performances to a combination of interrelated factors such as the distance of the Ru atoms to the dopped rGO support and the hydride content at the Ru NP surface.

## Graphical abstract

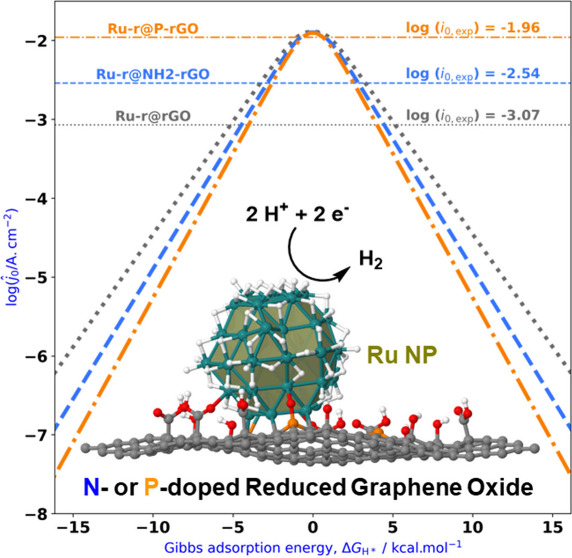

## 1 Introduction

Climate change has become one of the main concerns of humanity. One way to tackle this issue is the use of renewable H_2_ as a clean energy vector. This can be achieved using electrocatalysts able to reduce protons into hydrogen, with the help of green electricity, in the so-called hydrogen evolution reaction (HER).^1^

Graphene (G) possesses high surface area, stability and electronic conductivity, making it suitable for its use in electrocatalysis.^2^ In addition, its electrocatalytic activity can be tuned by doping its structure with heteroatoms, such as N,^3–6^ P,^4–7^ S,^5,8^ O,^5^ B^5^ and F,^5^ where it has been observed by DFT calculations that N and O become negatively charged (electron acceptors for adjacent C), while B, S, P and F become positively charged (electron donors).^5^

Given the well-known electrocatalytic activity of heteroatom doped-G systems, many researchers have used these materials as supports to study the HER electrocatalytic activity of metallic nanoparticles (NPs). By taking advantage of the putative synergistic electronic transfer effects between the supports and the NPs, it is expected to obtain higher HER activities and lower η_10_ values. Furthermore, the use of G-based supports allows attaining more stable and better dispersed NPs by restraining their aggregation.^9,10^

Among the different metallic NPs of interest for HER, Ru has emerged in the past decade as an attractive option due to its almost optimal ruthenium–hydrogen bond strength and its elevated stability in the whole pH range.^11,12^ The literature reports several examples with interesting low η_10_ values at acidic pH: 29 mV and 15 mV for 1.9 and 1.5 nm Ru NPs deposited on alginate-derived G and P-doped G, respectively, after a reductive activation process (Ru/G-r and Ru/P-G-r, respectively),^13^ 55 mV for a 3.5 nm Ru NP-modified N-doped G aerogel (Ru-NGA),^14^ 62 mV for 2.1 nm Ru NPs embedded in N-doped carbon (Ru@NC),^15^ 126 mV for 2–3 nm Ru NPs supported on N-doped carbon (Ru@CN)^16^ and 53 mV for 3–7 nm Ru nanoclusters deposited at 750 °C on top of N-doped G (Ru/NG-750).^17^

Interestingly, some studies combining experimental electrochemical data and DFT calculations dealing with the effect of the interaction between Ru NPs and a doped-G structure to tune the overall HER electrocatalytic activity have been recently published.^18–20^ Most of these studies intrinsically rely on the calculation of the hydrogen adsorption free energy, Δ*G*_H*_, a property considered as a descriptor for the HER in the framework of the seminal Nørskov electrochemical model.^21,22^ From those, it has been demonstrated by Δ*G*_H*_ calculations, density of states (DOS) studies and charge distribution analysis that the presence of O atoms due to the oxidation of porous graphene reduces the Δ*G*_H*_ value for the G-supported Ru NPs.^19^ Furthermore, it has been shown by Δ*G*_H*_ calculations, Bader charge computation and crystal orbital Hamilton population analysis that Ru NPs bind through a stable Ru–O–C bond to a reduced graphene oxide (rGO).^20^ This Ru–O bond induces an electron deficiency on the Ru NPs, which in turn reduces the barriers for the HER, making Δ*G*_H*_ closer to 0 kcal·mol^–1^ and the Ru–Ru bonds in the NP stronger. Also, it has been demonstrated by kinetic Monte Carlo (KMC) simulations that for strong or moderate H-binding metals (such as Ru) containing a high amount of adsorbed H atoms, there is a spillover of these H atoms from the NPs surface to the rGO surface that is beneficial for the HER process in alkaline media, diminishing the overpotentials.^23^ Finally, other approaches combining different levels of theory have been recently used to study the mechanisms (kinetic properties) and mass transport phenomena induced by metallic systems deposited onto C-based or semiconductor supports.^24–26^ For instance, *ab initio* molecular dynamics (AIMD) simulations have been employed to study the H_2_ adsorption capacity and spillover effect of Pd_4_ and Pd_3_P clusters anchored on different G-surfaces.^24^ However, to the best of our knowledge, these approaches have not yet been applied to Ru/rGO systems for HER electrocatalysis.

Additionally, Ru_2_P NPs have also been deposited onto non-doped rGO^27^ and N,P-*co*-doped C nanofibers,^28^ achieving very competitive HER electrocatalytic activities at acidic pH (η_10_ as low as 22 mV and 15 mV, respectively). For the former, according to DFT calculations, partial electron transfer from the Ru_2_P NPs to the surface sp^2^-C atoms of rGO favors hydrogen adsorption and recombination,^27^ and for the latter, the superior HER performance of the NPs is associated with the modification of their Δ*G*_H*_ by the N,P-*co*-doped C surface, which boosts their proton adsorption and reduction capacities.^28^ Thus, a positive effect in the HER electrocatalytic activity of Ru_2_P NPs has been demonstrated with N,P-*co*-doped C-based supports. But, to the best of our knowledge, there is no literature report in which metallic Ru NPs have been deposited independently on top of N-doped and P-doped rGO supports and their differential HER electrocatalytic behavior studied both from the experimental and theoretical points of view in comparison with the non-doped rGO counterpart.

In this work, we study the effect of N- and P-doping of reduced graphene oxide in the HER electrocatalytic performance of rGO-deposited Ru NPs by using a full set of experimental ((HR)­TEM, EDX, EA, ICP-OES, XPS, Raman, NMR, and LSV) and theoretical (DFT analysis) tools. The NPs have been deposited on-top of the preformed rGO supports by slow decomposition of a Ru organometallic complex under hydrogen pressure,^29^ allowing to obtain surface clean Ru NPs. Additionally, the use of rGO as a support decreases the concentration of oxygen-based groups at the surface, which may block the interaction between the organometallic precursor and the graphitic structure and prevent the formation of the NPs, as has recently been observed by some of us.^30^ Furthermore, in contrast to the frequent practice of synthesizing the rGO and the Ru NPs simultaneously by chemical reduction, the method used here allows fine-tuning the heteroatom-doping of the rGO prior to its use as a support. Finally, by using the experimental EA, XPS, and Raman data of the supports we have built up realistic rGO theoretical surfaces onto which DFT calculations (d-band center and charge analysis, Δ*G*_H*_ determination) have been performed with two different Ru NP models. Volcano plots have been calculated in the framework of the Yang, Saidi, and collaborators electrochemical model,^31,32^ which is an improvement of the Nørskov model.^21,22^ Both provide valuable theoretical framework for understanding the kinetics and mechanisms of HER. The variables that affect the electrochemical HER activity of the rGO-supported Ru NPs, such as the distance of the Ru atoms to the support or the hydride content of the Ru NP surface, have also been analyzed.

## 2 Materials and Methods

### 2.1 Chemicals, Solvents, and General Synthetic Procedures

All chemical operations were carried out under inert atmosphere by using Schlenk flasks, Fischer–Porter reactors, vacuum-line techniques and a glove box (Vigor). THF (Merck) was purified and deoxygenated before use by distillation under argon atmosphere through filtration in the column of a solvent purification system (SPS) and was degassed by freeze–pump–thaw process (x3) prior to use. The metal precursor [Ru­(COD)­(COT)] (COD = 1,5-cyclooctadiene, COT = 1,3,5-cyclooctatriene) was purchased from NanoMePS (NanoMatériaux en Poudres et Solutions, Toulouse).

### 2.2 Synthesis of rGO, NH_2_-rGO, P-rGO and Ru@x-rGO (x = none, NH_2_, or P)

rGO was synthesized as described elsewhere^30^ from GO prepared according to the modified Hummers’ method.^33^ Elemental analysis: C = 83.6 wt%, H = 0.5 wt%, S = 0.14 wt%.

NH_2_-rGO was prepared following a previously reported synthetic method.^3,30^ Elemental analysis: N = 7.91 wt%, C = 75.43 wt%, H = 1.69 wt%.

P-rGO was prepared as previously described.^4,7^ Elemental analysis: C = 85.0 wt%, H = 0.50 wt%. P = 2.94 wt% was determined by XPS analysis.

Ru@rGO and Ru@NH_2_-rGO. Both metal/support materials were prepared following the previously reported synthetic method.^30,34^ **Ru@rGO**: mean size = 2.0 ± 0.8 nm, ICP-OES = 2.4 wt% Ru. **Ru@NH**_**2**_ **-rGO**: mean size = 1.5 ± 0.2 nm, ICP-OES = 2.5 wt% Ru.

Ru@P-rGO. A Fischer–Porter reactor containing a suspension of P-rGO (100 mg) in 50 mL of THF was sonicated for 90 min. Then, a solution of [Ru­(COD)­(COT)] (10 mg, 0.032 mmol) in 3 mL of anhydrous and deoxygenated THF was transferred to the Fischer–Porter, and the reactor was pressurized with 3 bar of H_2_ while stirred vigorously for 20 h at room temperature (RT). Afterward, the remaining H_2_ pressure was released and the Ru@P-rGO material was recovered from the suspension by filtration through a polyamide membrane (Whatman membrane filters, 47 mm × 0.45 μm) and washed with THF (50 mL). The resulting black precipitate was dried overnight at 60 °C. Ru@P-rGO: mean size = 1.5 ± 0.3 nm, ICP-OES = 3.1 wt % Ru.

### 2.3 Characterization Techniques

Transmission electron microscopy (TEM) and high-resolution TEM (HRTEM): All materials were analyzed before and after catalysis by TEM and HRTEM at the “Servicio de Microscopía Electrónica” of the Universitat Politècnica de València (UPV) and “Servei de Microscòpia de la UAB”. Specifically, TEM analysis was performed by using a JEOL JEM 1400 Flash electron microscope operating at 120 kV with a point resolution of 3.8 Å, and HRTEM measurements were performed using a JEOL JEM 2010 electron microscope working at 200 kV with a resolution point of 2.35 Å. Size distributions were determined through manual analysis of enlarged micrographs with ImageJ software by counting over than 200 particles.

Elemental Analysis (EA): EA analyses were performed in a Euro EA3000 elemental analyzer (EuroVector) employing sulfanilamide as standard.

Inductively coupled plasma optical emission spectroscopy (ICP-OES): ICP-OES analyses of **Ru@rGO**, **Ru@NH**_**2**_ **-rGO** and **Ru@P-rGO** were performed at ITQ by using a Varian 715-ES ICP-Optical Emission Spectrometer, in order to determine the Ru contents. The samples were prepared following a modified digestion method previously reported:^35^ 30 mg of each sample were suspended in 21 mL HCl-HNO_3_ (6:1). Then, the solutions were sonicated for 90 min and digested at 180 °C for 15 h. Finally, they were cooled down up to RT and diluted with 100 mL of water prior to analysis by ICP-OES.

Raman spectroscopy: Raman spectra were recorded with a 514 nm laser excitation in a Renishaw in a Raman spectrometer equipped with a Lyca microscope. The samples (powders) were deposited on an Al support and measured in the region between 0 and 3000 cm^–1^ with a resolution of <4 cm^–1^.

Solid-state MAS NMR spectroscopy: ^31^P solid-state MAS NMR spectroscopy analysis was performed at the ITQ on a Bruker Avance 400WB instrument equipped with a 4 mm probe with the sample rotation frequency of 10 kHz. Measurements were carried out in a 4 mm ZrO_2_ rotor.

X-ray photoelectron spectroscopy (XPS): XPS analyses of **Ru@rGO**, **Ru@NH_2_-rGO** and **Ru@P-rGO** were performed using a SPECS device equipped with a Phoibos 150–9MCD detector using Al–Kα radiation (*h*ν = 1483.6 eV) with a pass energy of 30 eV. The pressure during the measurements was kept under 10^–9^ Torr. The quantification and evaluation of the spectra were done with the help of the CASA software, referencing them in base of *C*_1*s*_ = 284.5 eV. In order to study the evolution of the Ru oxidation state of the three Ru-containing materials, these were treated under H_2_ at 180 °C for 5 h, as already described elsewhere.^30^

### 2.4 Electrochemical Measurements

Electrodes were prepared as follows: a dispersion of 2 mg·mL^–1^ was prepared by adding 1 mg of each material in 500 μL of THF. Sonication was then applied to obtain a homogeneous dispersion and avoid material aggregation, and an aliquot of 5 μL was drop-casted on the surface of a glassy carbon rotating disk electrode (GC-RDE, Ø= 0.3 cm, *S* = 0.07 cm^2^) and dried out. This procedure was repeated three times. A 5 μL-drop of Nafion polymer (0.02 wt %) was added to the bare rGO and the **Ru@rGO** material and dried prior to the measurements.

Electrochemical measurements were performed at RT on a BioLogic SP-150 potentiostat with a GC-RDE working at 3000 rpm to ensure complete removal of *in situ* formed H_2_ bubbles. The solutions were degassed before the electrochemical analysis with an N_2_ flow. Ohmic potential (IR) drop was automatically corrected at 85% using the Biologic EC-Lab software for linear sweep voltammetry (LSV), cyclic voltammetry (CV) and chronoamperometry (CA). Chronopotentiometry (CP) experiments were performed under constant current density of −10 mA·cm^–2^ and IR drop was manually corrected (*E*_mod_ = *E*_meas_ + *E*_IR_, mod = modified and meas = measured) at 85% by adding the corresponding potential value *E*_IR_ = *i*_exp_ × (*R*_mes_ *x 0.85)*, where *i*_exp_ is the applied current in A and *R*_mes_ is the measured resistance in Ω. One M H_2_SO_4_ solution was prepared by mixing 56.1 mL of 95–97% H_2_SO_4_ in 1 L of Milli-Q water. The measurements were performed in a three-electrode configuration using a saturated calomel electrode (SCE, Hg/Hg_2_Cl_2_, KCl sat.) and a Pt wire as reference (RE) and counter (CE) electrodes, respectively. The GC-RDE was used as working electrode (WE) after drop-casting each sample from a THF suspension (2 mg/mL). All catalytic experiments were measured with a sweep rate of 10 mV·s^–1^. Potentials are reported vs RHE (*E*_RHE_ = *E*_SCE_ + 0.244 V + (0.059 × pH)) and overpotentials for HER at a current density of 10 mA·cm^–2^ are calculated as η_10_ = (0–0.059 × pH) – (*E*_SCE_ + 0.244 V).

A 10 mL proton exchange membrane-separated two-compartment cell was used for the Faradaic efficiency calculation. The CE was placed in one compartment and the WE and RE were placed in the other compartment together with the Clark electrode. Both compartments were filled with 7 mL of 1 M H_2_SO_4_ solution and equipped with a stirring bar. Prior to each measurement, both compartments were purged with N_2_. A H_2_–NP Clark electrode (Unisense) was used to measure in the gas phase the hydrogen generated by the materials during the chronopotentiometry (bulk electrolysis). The Clark electrode was calibrated by adding different volumes of 99% pure hydrogen at the end of each experiment.

The double-layer capacitance (*C*_DL_) of each material was determined as follows: *C*_DL_ was estimated by recording CVs in a non-faradaic region (typically a 0.1 V window about OCP), where no redox processes take place, and all the measured current is due to double-layer charging. In this region, 8 different scan rates were used (5, 10, 25, 50, 75, 100, 250, and 500 mV·s^–1^), holding the WE at each potential vertex for 10 s prior to the next step. Then, the determination of the Electrochemically Active Surface Area (ECSA) and the Tafel plot analysis of each material were achieved as described elsewhere.^36^ The cathodic transfer coefficient (α) was determined by assessing the slope of the linear part of the plot of −(*R*·*T*/*F*)·ln|*j*|(A·m^–2^) vs. *E*(V).

## 3 Computational Details

### 3.1 Energy Calculations

Density functional theory (DFT) in periodic boundary conditions calculations were performed using the *ab initio* plane-wave pseudopotential approach, as implemented in the Vienna Ab initio Simulation Package (VASP; version 5.4).^37,38^ The Perdew–Burke–Ernzerhof exchange–correlation functional^39^ within the generalized gradient approximation was chosen, and van der Waals interactions were taken into account through the D3 method of Grimme et al. with zero-damping function.^40^ The innermost electrons were replaced by a projector-augmented wave (PAW) approach,^41,42^ while the valence monoelectronic states were expanded in a plane-wave basis set with a cutoff energy of 450 eV. Partial occupancies were estimated with a Gaussian smearing (σ) of 0.05 eV during all relaxations and extrapolating the energies to σ = 0.00 eV. The model carbon supports were designed as a graphene slab, showing that a (10 × 10) supercell was found to be large enough to include a broad representation of experimentally probed functional groups. This unit cell contains 200 carbon atoms, and a Γ-centered (3 × 3 × 1) *k*-point grid generated using the Monkhorst–Pack method^43^ was employed as a good compromise between accuracy and computational cost. All the systems considered for the present study are neutral. Spurious interactions between the modeled slab and its periodic images perpendicular to the slab were eliminated by introducing a vacuum region by at least 10 Å and by applying a dipole correction to the total energy along the *z*-direction.^44^ Iterative relaxation of atomic positions proceeded until the change in total energy between successive steps was less than 10^–6^ eV per cell, and the residual forces in any direction acting on the atoms were less than 0.015 eV Å^–1^.

### 3.2 Molecular Dynamics

To study the thermal stability of carbon-based materials, first-principle Born–Oppenheimer *ab initio* molecular dynamics (AIMD) simulations were performed within the *NVT* statistical ensemble (canonical ensemble), *i*.*e*., under the conditions of a constant particle number, constant volume, and a temperature fluctuating around an equilibrium value. Starting from the optimized structure, each slab was propagated at 450 K (temperature at which supports were experimentally synthesized) for 10 ps at time steps of 0.5 fs for integrating the atomic equations of motion. The level of theory was the same as described above, and the *k*-mesh was reduced to a gamma point. In all cases, the electronic structures were well-converged to 10^–6^ eV. The temperature was regulated using the Nosé thermostat.^45^ This procedure has been applied to the rGO model and to its N-doped and P-doped counterparts. The geometry obtained at the end of each AIMD simulation has then been optimized without any symmetry constraint in order to provide a model used all throughout the DFT study of the HER at the surface of Ru NPs supported on rGO surfaces.

### 3.3 Electronic Structure Analysis

To provide additional insights on the modeled supports, as well as the interaction between the Ru nanocatalysts and the support, density of states (DOS) and crystal orbital Hamilton population (COHP) analysis^46^ were performed with the LOBSTER package.^47,48^ Based on contributions from orbital pairs, COHP is an energy division of the band structure, so it acts on a local basis. In this regard, from a plane-wave DFT output, the LOBSTER package allows calculating the projected COHP in a local atomic Slater functions basis set (pCOHP) as well as a consistent density of states projected by atomic orbitals (pDOS). For all the compounds studied in this work, the charge spilling, a measure that assesses the quality of projection in the chosen pbeVASPfit basis set, reaches a maximum value of 2%. A Mulliken population analysis (MPA) can be obtained by integrating the atom-selected pDOS up to the Fermi energy. All MPA calculations and assistance to pDOS and pCOHP analysis were carried out with an in-house tool, selectLOBSTER.^49^ The COHP bond index used to support the discussion is described in section 2.4 of the Supporting Information (SI) and has been calculated by integrating a given COHP profile up to the Fermi energy.

## 4 Results and Discussion

### 4.1 Synthesis and Characterization

The **Ru@rGO** and **Ru@NH**_**2**_ **-rGO** cathodic materials were previously synthesized by some of us for their use in catalytic processes other than HER.^30,34^ We have enlarged this group of catalysts to the **Ru@P-rGO** counterpart to perform a full experimental and theoretical comparative study of the effect of independent N- and P-doping of rGO in the HER electrocatalytic activity of the respective doped and undoped rGO-supported Ru NPs. **Ru@P-rGO** was prepared following the same synthesis protocol as for **Ru@rGO** and **Ru@NH**_**2**_ **-rGO** (Scheme 1).^30,34^ Thus, the decomposition of [Ru­(COD)­(COT)] under mild conditions (3 bar H_2_, 20 h, RT) over the three supports led to the formation of small Ru NPs. The metal contents of the three materials were determined by ICP-OES, providing in all cases values close to the theoretical one, 3 wt %, *i*.*e*., **Ru@rGO**, 2.4 wt% Ru; **Ru@NH**_**2**_ **-rGO**, 2.5 wt% Ru; and **Ru@P-rGO**: 3.1 wt% Ru.

**Scheme 1:**
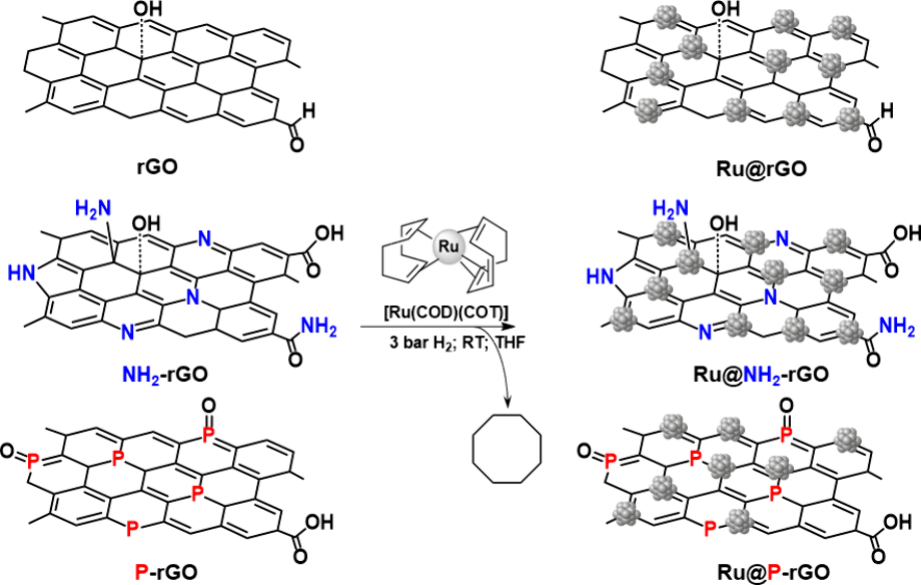
Synthesis of **Ru@rGO**, **Ru@NH**_**2**_ **-rGO**, and **Ru@P-rGO** Materials from the [Ru­(COD)­(COT)] Organometallic Complex (Grey Spheres Represent Ru Atoms)

Transmission electron microscopy (TEM) analyses revealed the formation of small, monodisperse and well-distributed NPs in all cases. **Ru@NH**_**2**_ **-rGO** and **Ru@P-rGO** show the smallest and most homogeneous particle size with a mean diameter of ca. 1.5 nm (SI, Figure S1d–i), with low size dispersion (±0.2 and ±0.3 nm, respectively) and good distribution onto the carbonaceous support. The undoped rGO material, **Ru@rGO**, presents higher particle mean size and size dispersion (2.0 ± 0.8 nm) and worse distribution than its doped counterparts (SI, Figure S1a–c). These differences in Ru NP size homogeneity and distribution result from the positive effect of the heteroatom doping in the stabilization of Ru NPs and distribution at the rGO surface, as previously observed in similar doped graphene systems.^13,30,34^ High resolution TEM (HRTEM) analyses confirmed the crystallinity of the Ru NPs in all cases (SI, Figure S2). As previously observed for **Ru@NH**_**2**_ **-rGO**,^30^ the Fourier analysis of the HRTEM image of **Ru@P-rGO** indicates the presence of a hexagonal close packed (hcp) structure typical for Ru^0^ (active species in H_2_ dissociation), with reflections due to the (100) and (002) atomic planes (SI, Figure S2c).

P-rGO and **Ru@P-rGO** materials were analyzed by Raman spectroscopy. As in the rGO^50^ and NH_2_-rGO^30^ spectra, the spectrum of P-rGO exhibits two major bands at 1368 cm^–1^ (D peak) and 1592 cm^–1^ (G peak), together with a broad peak around 3000 cm^–1^ (2D peak) (SI, Figure S3a). The high D/G ratio (*I*_D_/*I*_G_ = 2.01) is related to the large percentage of defects of this support, which are excellent anchoring points for NP stabilization, as compared to the previously reported *I*_D_/*I*_G_ ratios for rGO (1.54)^50^ and NH_2_-rGO (1.49).^30^ The presence of Ru NPs onto P-rGO (**Ru@P-rGO**) leads to a slight decrease in the *I*_D_/*I*_G_ ratio down to 1.92 (SI, Figure S3b), indicating a decrease in the number of defects in the P-rGO support due to the preferential growth of Ru NPs over these defects, improving their stability during electrocatalysis (*vide infra*).

The presence/nature of P atoms and the chemical composition of **Ru@P-rGO** were analyzed by X-ray photoelectron spectroscopy (XPS). The C 1s signal of P-rGO shows a peak at a binding energy (BE) of 284.8 eV, that can be deconvoluted into three components (SI, Figure S4c). The main component at 284.8 eV (red) belongs to the sp^2^ C atoms of the graphitic domains (∼77%), and the two minor contributions are attributed to carbon atoms linked to P or O atoms (287.0 eV; green, ∼11%) and to carboxylic groups (289.2 eV; blue, ∼12%). C 1s signals of rGO and NH_2_-rGO are very similar, with the only difference that the component at ∼286–287 eV (green) corresponds to carbon atoms of epoxides, tertiary alcohols and/or C atoms connected to N (SI, Figure S4a,b). The presence and nature of P atoms on the P-rGO support were confirmed after analyzing the P 2p region (SI, Figure S5a). The P 2p area shows a broad peak which can be deconvoluted in two contributions, one centered at 133.1 eV (gray), corresponding to P atoms bonded to oxygen atoms (∼41%), and another one at 130.9 eV (blue), attributed to P–C bonds (∼59%). These peaks have been previously identified in similar P-doped graphenes.^51,52^ In the P-rGO case, the percentage of graphitic phosphorus atoms (P–C) is higher than in the P-doped graphene obtained from alginate,^13^ where P is mainly in the phosphate (P–O) form. The existence of this minor (∼41%) P–O phase in P-rGO indicates that the doped P atoms have been partially oxidized by the oxygen released from GO during the synthetic reductive process. Analysis of the P 2p signal allowed to determine the P content of P-rGO, which is 1.26 at % (2.94 wt %). Also, as previously reported,^30^ the N-containing groups present in NH_2_-rGO are graphitic, pyridinic, pyrrolic, or amino groups, the last two being the most abundant ones.

Moreover, the oxidation state of Ru in **Ru@P-rGO** before and after reduction treatment (180 °C under an H_2_ flow for 5 h) was studied by XPS analysis of the Ru 3p region. SI, Figure S5b, exhibits the Ru 3p_3/2_ area of the as-synthesized **Ru@P-rGO**, where a peak centered at 463.2 eV is observed. The deconvolution of this peak presents two different components, one at 464.4 eV, attributed to Ru­(IV) and characteristic of RuO_2_, and another one at 462.7 eV, corresponding to Ru(0).^36^ These results confirm the formation of a RuO_2_ passivation layer around the Ru(0) core after purification of the material in air, as we have otherwise demonstrated in related systems.^12,36^ However, after applying reduction conditions, the Ru(0) component at ca. 462 eV increases at the expense of that corresponding to RuO_2_ (SI, Figure S5c). In the same way, the Ru 3p XPS spectra of **Ru@rGO** and **Ru@NH**_**2**_ **-rGO** show that before reduction treatment most of the Ru is as RuO_2_, whereas after the reduction treatment the main component is Ru(0) (SI, Figure S6). Precise amounts of Ru(0) and RuO_2_ before and after reduction treatment are shown in SI, Table S1, indicating higher percentages of Ru(0) after reduction in the doped materials than in the undoped one (48.4% for **Ru@rGO**, 72.6% for **Ru@NH**_**2**_ **-rGO** and 87.3% for **Ru@P-rGO**).

Finally, the nature of the P atoms in P-rGO has also been examined by ^31^P MAS solid state NMR spectroscopy. The ^31^P MAS NMR spectrum shows a very broad band centered at 0 ppm (SI, Figure S7). According to the literature, the width of this peak is attributed to a wide array of sites with slightly different isotropic chemical shifts,^4,53^ indicating the existence of graphitic phosphorus or oxidized graphitic phosphorus bonded to three sp^2^-hybridized carbon atoms, as has also indicated by the XPS studies.^4,53^

### 4.2 Electrocatalytic performance in the HER

The HER electrocatalytic performance of **Ru@rGO**, **Ru@NH**_**2**_ **-rGO**, and **Ru@P-rGO** was evaluated in 1 M H_2_SO_4_ aqueous solution. Working electrodes were prepared by dispersing the materials in THF (2 mg·mL^–1^) and drop-casting them onto a glassy carbon rotating disk electrode (GC-RDE). The polarization curves and corresponding Tafel plots of **Ru@rGO**, **Ru@NH**_**2**_ **-rGO**, and **Ru@P-rGO** are shown in Figure 1 (bold lines). A change in the current density is observed when scanning into reductive potentials, confirming the catalytic reduction of protons into H_2_.

**Figure 1:**
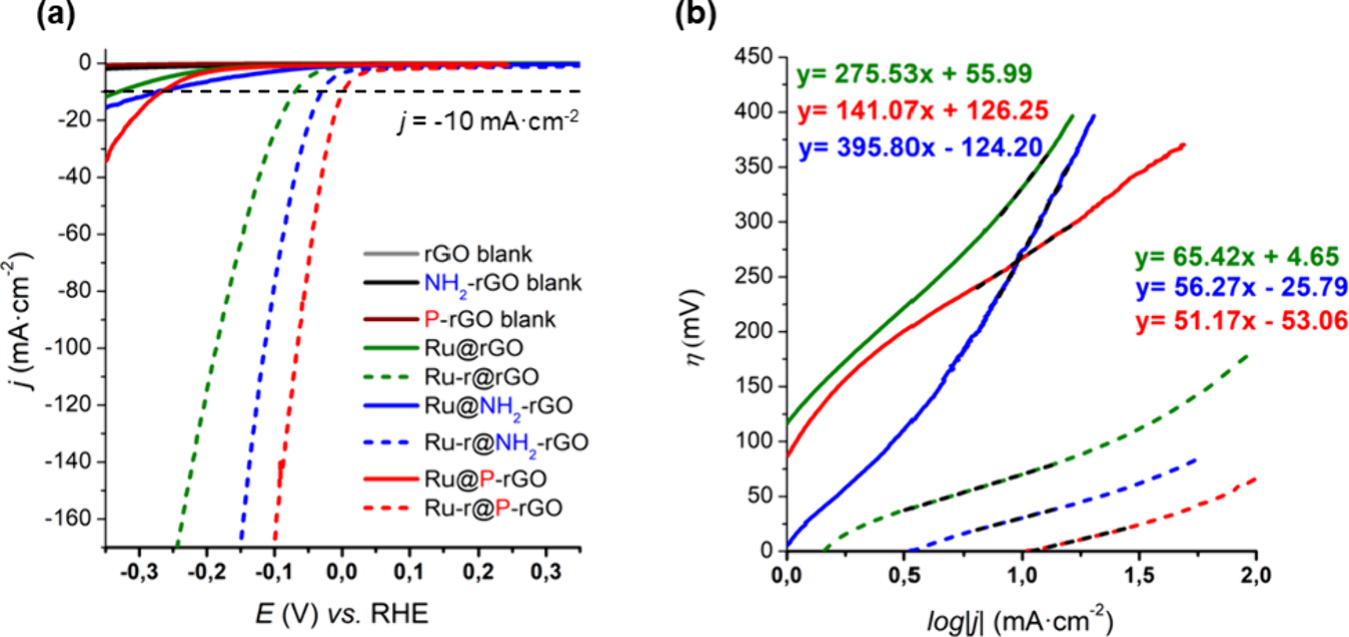
(a) Polarization curves of **Ru@rGO** (green line), **Ru@NH**_**2**_ **-rGO** (blue line), and **Ru@P-rGO** (red line) before (solid) and after (dashed) a 7–15 h reductive process at *j* = −10 mA·cm^–2^ in 1 M H_2_SO_4_. rGO (gray line), NH_2_-rGO (black line), and P-rGO (wine line) blanks are also shown. (b) Tafel plots of **Ru@rGO**, **Ru-r@rGO**, **Ru@NH**_**2**_ **-rGO**, **Ru-r@NH**_**2**_ **-rGO**, **Ru@P-rGO**, and **Ru-r@P-rGO** in 1 M H_2_SO_4_. Same color code as in (a).

The catalytic performance of the materials can be significantly improved when submitted to a 7–15 h current-controlled bulk electrolysis at *j* = −10 mA·cm^–2^. As presented in Figure 1a, whereas **Ru@rGO**, **Ru@NH**_**2**_ **-rGO**, and **Ru@P-rGO** show a η_10_ of 331 mV, 274 mV and 268 mV, respectively, a shift in the polarization curves is observed after the reductive process, decreasing the η_10_ down to 71 mV, 30 mV and 2 mV, respectively. This behavior is attributed to a change in the oxidation state of surface Ru atoms on the NPs. As shown by the XPS data (*vide supra*) and our related studies,^12,36^ the Ru NP surface gets partially oxidized (passivated) when exposed to air after their synthesis. When submitted to a reductive potential, this RuO_2_ passivated surface is reduced back to metallic Ru. Thus, the catalytic current density of the reduced materials, from now on **Ru-r@rGO**, **Ru-r@NH**_**2**_ **-rGO**, and **Ru-r@P-rGO**, radically increases in comparison with that of the passivated materials (*i*.*e*. **Ru@rGO**, **Ru@NH**_**2**_ **-rGO**, and **Ru@P-rGO**, Figure 1a). We previously performed a deep study of this behavior on 4-phenylpyridine (4-PP) capped^36^ and carbon nanotubes- supported Ru (Ru/RuO_2_) NPs.^12^ In both cases, a total disappearance of the RuO_2_ peak in the XPS was observed, indicating the reduction of the superficial Ru­(IV) into Ru(0) under reductive electrochemical catalytic conditions, which can be seen as an activation step.

The different electrocatalytic performance of the as-synthesized/activated **Ru@rGO**/**Ru-r@rGO**, **Ru@NH**_**2**_ **-rGO**/**Ru-r@NH**_**2**_ **-rGO**, and **Ru@P-rGO**/**Ru-r@P-rGO** samples is evidenced from their corresponding Tafel plots (Figure 1b). **Ru-r@rGO** shows a lower Tafel slope of 65 mV·dec^–1^ compared to 276 mV·dec^–1^ for **Ru@rGO**. The same tendency is observed with the heteroatom-doped materials, with **Ru-r@NH**_**2**_ **-rGO** showing a slope of 56 mV·dec^–1^ vs. 396 mV·dec^–1^ for **Ru@NH**_**2**_ **-rGO**, and **Ru-r@P-rGO** a slope of 51 mV·dec^–1^ vs. 141 mV·dec^–1^ for **Ru@P-rGO**, as expected for the presence of Ru(0) species in the activated materials by bulk electrolysis. A positive effect of the N- and P-doping rGO supports is also observed in the HER catalytic activity. **Ru-r@NH**_**2**_ **-rGO** and specially **Ru-r@P-rGO** show lower overpotentials and Tafel slopes than their bare counterpart **Ru-r@rGO**, and represent some of the best Ru-based HER electrocatalysts reported so far in the literature (see SI, Table S2). As a matter of fact, our activated P-doped material shows the lowest η_10_ value reported until now (only 2 mV).

The Tafel slope (*b*) allows defining the rate-determining step (rds) of the HER. **Ru-r@rGO**, **Ru-r@NH**_**2**_ **-rGO**, and **Ru-r@P-rGO** show slopes of 65, 56, and 51 mV·dec^–1^, respectively (Figure 1b), suggesting that the HER follows a mechanism in between the Volmer and Heyrovsky steps as rds. In contrast, **Ru@P-rGO** shows a Tafel slope close to 120 mV, typically attributed to the Volmer step (adsorption of H^+^ to form the M–H species) as the rds, whereas **Ru@rGO** and **Ru@NH**_**2**_ **-rGO** show very big Tafel slopes, suggesting an extremely slow H^+^ adsorption step related to the presence of a poor catalytically active RuO_2_ shell in the as-prepared samples.

The fate of the materials after short-term stability tests (2 h) was assessed by TEM analysis. After 2 h under catalytic conditions at a constant *j* of −10 mA·cm^–2^, each drop-casted material was recovered from the GC-RDE with THF and sonication. Then, a drop of the THF suspension of the recovered material was deposited onto a carbon-coated copper TEM grid. As shown in SI, Figure S8, the mean size of the Ru NPs significantly increased for **Ru@rGO**, while it was similar for **Ru@NH**_**2**_ **-rGO** and **Ru@P-rGO** compared to the corresponding initial materials. These results support the hypothesis that heteroatoms (*i*.*e*. N or P) stabilize Ru NPs from aggregation/coalescence both during the synthetic process (due to the high number of defects present in the NH_2_-rGO and specially the P-rGO surface according to Raman spectroscopy, *vide supra*) and under electrocatalytic conditions.

Long-term stability is a key parameter for a HER electrocatalyst to attain practical use. Thus, once activated, the **Ru-r@rGO**, **Ru-r@NH**_**2**_ **-rGO**, and **Ru-r@P-rGO** electrodes were held at a constant current density of *j* = −10 mA·cm^–2^ in a current controlled experiment (*i*.*e*. chronopotentiometry) for 12 h, monitoring the change in the required overpotentials (SI, Figure S9). LSVs before and after the 12 h experiments were also performed and are also shown in SI, Figure S9. The **Ru-r@NH**_**2**_ **-rGO** and **Ru-r@P-rGO** materials show almost no change in η_10_ and almost identical LSV polarization curves before and after electrocatalytic turnover (SI, Figure S9b and S9c). For **Ru-r@rGO**, the stability is also confirmed, obtaining similar performance after 12 h under catalytic turnover. Therefore, even if **Ru@rGO** suffers aggregation and coalescence of Ru NPs during the activation process (see TEM images after 2 h in SI, Figure S8), the resulting **Ru-r@rGO** nanomaterial presents stable HER electroactivity for at least 12 h. Altogether, the elevated stability of the three supported systems upon un-interrupted catalytic turnover conditions may be related to the strong coordination energies of these Ru NPs onto the supports (see DFT calculations below). Also, faradaic efficiencies of 96–98% were determined by quantifying the amount of H_2_ generated during a 20 min chronoamperometry using a H_2_–Clark electrode and comparing it with the maximum theoretical amount of H_2_ calculated from the total charge passed through the electrode (SI, Figure S10). This confirms the production of H_2_ as the sole reaction occurring with all materials studied.

A final comparison of the main physicochemical and electrocatalytic data for the three studied materials is shown in Table 1, including the calculation of the exchange current density (*j*_0_) for all and of the cathodic transfer coefficient (α) for the activated materials only (SI, Figure S11). These data will be discussed and used for the theoretical modeling (*vide infra*).

**Table 1:** Summary of Physicochemical and HER Electrocatalytic Propertiesfor the Materials Studied (1 M H_2_SO_4_ Aqueous Solution at pH 0)^a^

entry	system	*d*_mean_*t* = 0 (nm)	*d*_mean_*t* = 2h (nm)	Ru (wt %)	η_10_ (mV)	*b* (mV·dec^–1^)	*j*_0_ (mA·cm^–2^)	α
1	Ru@rGO	2.0 ± 0.8	4.3 ± 0.9	2.4	331	276	0.63	
2	Ru-r@rGO				71	65	0.85	0.39
3	Ru@NH_2_-rGO	1.5 ± 0.2	1.9 ± 0.23	2.5	274	396	2.06	
4	Ru-r@NH_2_-rGO				30	56	2.87	0.45
5	Ru@P-rGO	1.5 ± 0.3	1.4 ± 0.2	3.1	268	141	0.13	
6	Ru-r@P-rGO				2	51	10.88	0.50

a Parameters: mean diameter (*d*_mean_) before (*t* = 0) and after (*t* = 2 h) catalysis, onset overpotential at −10 mA·cm^–2^ (η_10_), Tafel slope (*b*), exchange current density (*j*_0_), and cathodic transfer coefficient (α).

As discussed above, the activation of the materials provokes a concomitant decrease of the Tafel slope (*b*) and of the η_10_ value for all three materials, especially for the P-doped one, yielding a η_10_ value as low as 2 mV. Concerning the *j*_0_ value, it increases moderately for the rGO and NH_2_-rGO supports upon activation (ca. 35–40%), and dramatically for P-rGO (more than 80 times), reaching up to 10.88 mA·cm^–2^. Thus, the tendency of all electrochemical parameters clearly points to a **Ru-r@rGO** < **Ru-r@NH**_**2**_ **-rGO** < **Ru-r@P-rGO** trend in HER performance.

Finally, the electrochemically active surface area (ECSA) for the non-activated materials **Ru@rGO**, **Ru@NH**_**2**_ **-rGO**, and **Ru@P-rGO** has been assessed (SI, Figure S12), obtaining very similar values for all materials (5.9, 4.0, and 2.9 cm^2^, respectively), in contrast to previous results obtained for related alginate derived graphene,^13^ where the incorporation of P into the carbon structure made the final material more exfoliated and less rough. The similarity in ECSA of the three materials before activation here studied indicates that the differences observed among the three activated systems may not be due to a significant difference in the number of active sites, but to different intrinsic catalytic activities of the catalytic sites on each support. To further confirm this hypothesis, DFT calculations have been performed as follows.

### 4.3 DFT studies

After having characterized the electrocatalytic behavior of the three rGO-supported Ru materials, and in view of the already-known respective electron acceptor and electron donor effects of N and P atoms adjacent to C atoms in graphitic structures,^5^ the electronic effects of these doping-heteroatoms in the materials have been addressed by a thorough DFT theoretical approach. The presence of the heteroatoms could endeavor a concomitant effect in the electronic structure of the supported Ru NPs, thus provoking a synergistic effect between the Ru NPs and the doped-rGO supports, modulating the adsorption energy of reaction intermediates to enhance the HER catalytic activity of the Ru NPs. To this end, the three supports have been modeled by AIMD simulations after considering their experimental EA, XPS and Raman data, following the design procedure of Navarro-Ruiz et al.^54^ Details are given in section 2.1.1 and Figures S13 and S14 of the SI. In short, it consists in accommodating experimentally proven structural defects, performing a sequence of serial optimizations on the defective graphene, and functionalizing the carbon surface, starting with those found within the carbon lattice, followed by increasingly larger groups. Ten ps AIMD simulations have been made to probe the thermal stability of these functionalized surfaces, reached after approximately 5 ps. The final geometry of the simulation is then relaxed by a local geometry optimization. The moderate concentration of oxygenated groups induces a loss of planarity of rGO, giving rise to the formation of remarkable superficial corrugations. The N-doped support presents a higher density of functionalities and, therefore, a higher deformation of the carbon surface. It is worth to mention the increase in vacancy defects on the carbon lattice due to the presence of pyridine and pyrrole rings, including some protonated ones. Functionalization is the least significant on the P-rGO support, partly due to the low P incorporation in the graphene oxide. This leads to a higher concentration of pristine graphitic areas and therefore less rippling of the carbon system. After the support design, DFT calculations (d-band center and charge analysis, Δ*G*_H*_ determination) have been performed with two different Ru NP models to analyze the variables that may affect the electrochemical HER activity of the supported Ru NPs, such as the distance of the Ru atoms to the support or the hydride content of the NPs surface.

The two molecular models that have been considered are the two previously published Ru_55_H_53_ and Ru_55_H_70_ models,^49^ each of them being adsorbed onto the rGO, NH_2_-rGO and P-rGO supports obtained after AIMD simulations (Figure 2, and SI, Figure S15). Coordination energies of the Ru_55_H*_n_* NPs reported in this figure (red numbers, in kcal·mol^–1^) correspond to the reaction Ru_55_H_*n*_ + substrate → Ru_55_H_n_* (given the in situ growth of these NPs, the Ru_55_H*_n_* reactant geometry is the relaxed geometry of its Ru_55_H*_n_* * counterpart, *i*.*e*., with no hydrides lying at their bottom). All Ru NPs are strongly bound to the support, in the order NH_2_-rGO > rGO > P-rGO. Also, we can see that superficial hydrides have a destabilizing effect on the coordination energies of the Ru NPs onto the supports, since they are less strongly attached when their hydride content increases.

**Figure 2:**
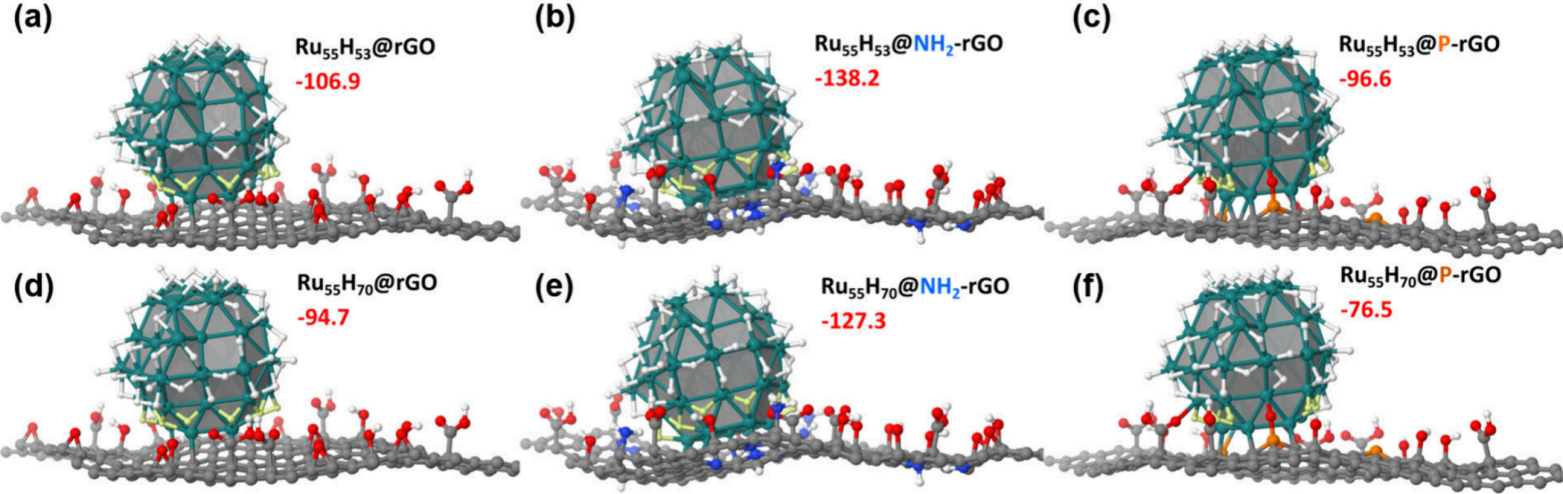
Side views of Ru_55_H_53_ and Ru_55_H_70_ NPs on the rGO (a,d), NH_2_-rGO (b,e), and P-rGO (c,f) functionalized supports (top views are given in SI, Figure S15). Atomic color scheme: Ru (green); P (orange); O (red); N (blue); C (gray); H (white). Gray surfaces highlight the faceting of the Ru_55_ hcp sphere. Yellow hydrogen atoms belong to the [0:3] Å domain, whose HER activity is discussed hereafter. The coordination energy of the Ru_55_H*_n_* NP is also given in each case, in kcal·mol^–1^ (red numbers).

The effect of each of the three supports on the electronic structure of the bare Ru_55_ model has been first evaluated to assess the intrinsic effect of the support on the electronic structure of the metallic Ru. It also provides clues about the adsorption strength of hydrides at the Ru NP surface, under the possible influence of the support. Yet, such bare NPs would tend to maximize their interaction with the support to compensate for the lack of stabilizing effect of surface hydrides. Besides, the geometry optimization leads to a significant increase of the interaction between some surface species of the rGO supports that is not representative of the actual interaction of these supports with hydrogenated Ru NPs. Therefore, this preliminary analysis was carried out on the Ru_55_H_70_@X frozen geometry, from which the 70 hydrides have been removed (X = rGO, NH_2_-rGO, P-rGO). After projection of the PAW wave functions into an auxiliary LCAO basis set by the Lobster software (see DFT computational details section in the SI), it is possible to calculate d-band centers^55^ and Mulliken-like population analysis (MPA).^56^ Results are summarized in Figure 3 (a detailed description of the values commented hereafter is given in the caption), in which the Ru NPs are discretized in a series of five parallel close-packed planes (cpp) numbered from 1 to 5, with cpp1 corresponding to the layer in contact with the support and cpp5 the outermost layer from the support (see SI, Figure S19, for a detailed description). Regarding the supports, unfunctionalized carbon atoms are in general neutral, while the functionalization involves small local charge transfer, as otherwise observed for small hydrogenated Pd NPs deposited on graphene-derived substrates.^54^

**Figure 3:**
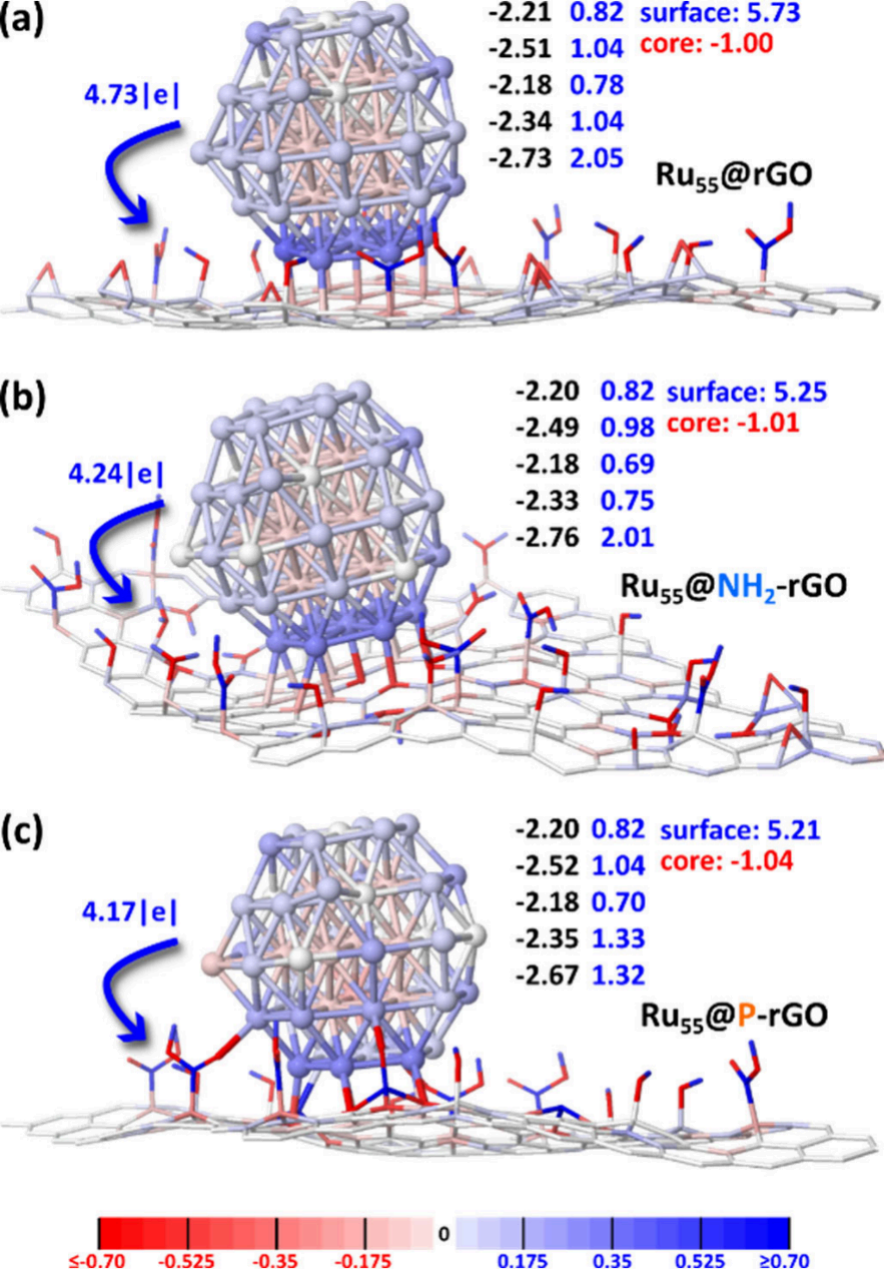
d-Band center and MPA charge analysis of Ru_55_@rGO (a), Ru_55_@NH_2_-rGO (b), and Ru_55_@P-rGO (c). Atom colors correspond to their electronic charge, according to the scale plotted at the bottom of the figure. rGO supports are plotted as wireframes to better see Ru atomic charges. The number above each blue arrow is the charge transfer from the Ru_55_ model to the support. The two-column numbers for each system are the average d-band center (left, in black) and the total charge (right, in blue) calculated for the surface Ru atoms of each of the five close-packed planes (cpp) from cpp1 (bottom) to cpp5 (top) (see SI, Figure S19, for a detailed description). Pay attention that the total charge of core Ru atoms is slightly negative (red numbers). The charge of surface atoms is also given.

The striking feature is the oxidation of the Ru_55_ model by all the supports, with a significantly stronger charge transfer induced by the rGO support (4.73 |*e*| vs. NH_2_-rGO, 4.24 |*e*|, and P-rGO, 4.17 |*e*|). The surface atoms of the close-packed plane (cpp) close to the support, cpp1, are in general more strongly oxidized than the other surface atoms (darker blue atoms and blue values at the column bottom in Figure 3). Whereas the MPA charge of cpp1 is ca. 2 |*e*| for Ru_55_@rGO and Ru_55_@NH_2_-rGO, it is only 1.3 |*e*| in Ru_55_@P-rGO. The reason probably lies in the direct interaction of surface species with one surface Ru atom of cpp2. The overall charge of surface atoms of cpp1 and cpp2 is similar between Ru_55_@NH_2_-rGO and Ru_55_@P-rGO (2.76 |e| and 2.65 |e|) and lower than the one calculated in the Ru_55_@rGO model (3.09 |e|). These local charges as well as the NP-to-support charge transfer are noticeable different features between Ru_55_@rGO on one hand and Ru_55_@NH_2_-rGO and Ru_55_@P-rGO on the other hand. Incidentally, they do not vary in the same order as the coordination energies of the Ru_55_H_n_ NPs (Figure 2) and thus there seems not to be any relationship between the coordination energy of the Ru NPs onto the supports and the charge transfer processes taking place between them.

The d-band centers, ε_d_, of the surface atoms of the cpp planes are also reported in black in Figure 3. ε_d_ calculated for the cpp1 surface atoms (−2.73 eV, −2.76 eV, −2.67 eV) lie farther away from the Fermi energy level than the other cpps, in line with the stabilization of these atoms by the direct interaction with the support. The other values are closer to the Fermi energy band, and there is virtually no difference between the three models. The fact that the d-band center of cpp2 to cpp5 lie close to the Fermi level means that oxygen atoms will adsorb very strongly once these NPs are put in contact with air, pointing that the RuO_2_ passivation layer will be highly stable and thus difficult to be removed by electrochemical reduction (Figure 1, bold lines). This would explain why very long electrochemical activation times (7–15 h) are needed to fully activate the rGO-supported Ru NPs (Figure 1, dashes lines), in contrast to their 4-phenylpyridine (4-PP) capped counterparts,^36^ which become completely active in HER electrocatalysis after a short 20-min activation time. On the other hand, the only significant difference in the Ru_55_@P-rGO model compared to the other two models is the d-band center of the cpp1 surface atoms, which is closer to the Fermi level by 0.06 and 0.09 eV with respect to Ru_55_@rGO and Ru_55_@NH_2_-rGO, respectively. This suggests, given the usual interpretation of the d-band center descriptor, that this part of the surface will be slightly more reactive in Ru_55_@P-rGO. These data, together with a lower Ru NP-to-support charge transfer in this system as well as in Ru_55_@NH_2_-rGO (4.17 |e| and 4.24 |e|, compared to 4.73 |e| in Ru_55_@rGO), indicate that a difference in the catalytic activity of the Ru surface can be expected between the three supported Ru NPs. This is what is now going to be assessed differently by calculating hydrogen adsorption free energies, Δ*G*_H*_, of all hydrides present at the surface of the Ru NPs as a function of their distance to the support surface (see section 2.3 of the SI for further details). As explained in section 2.2 of the SI, each Δ*G*_H*_ value can be easily correlated with the theoretical exchange current density value (*ĵ*) corresponding to each reactive site, obtaining the so-called volcano plots.

To compute the dependence of Δ*G*_H*_ vs. the exchange current density we have used the experimental cathodic transfer coefficient values (α values) and the recently improved Yang and Saidi model^31,32^ instead of the most frequently used (and less realistic in the more strongly adsorbed hydride regime) Nørskov model^21,22^ (see SI, section 2.2 and Figures S16–S18 and Table S3, for further details). Then, the theoretical Δ*G*_H*_ values were calculated for all sites at a distance *R* from the support (see SI, section 2.3, for more information), and the corresponding volcano plots were generated considering the overall effect of all hydrides present on the Ru NPs (Figure 4a) or only the ones at a distance *R* below 3 Å (Figure 4b). Furthermore, two different Ru NP models, namely Ru_55_H_53_ (1.2 H^–^ per surface Ru atom, Figure 4, top) and Ru_55_H_70_ (1.6 H^–^ per surface Ru atom, Figure 4, bottom), were considered. The ca. 1.2:1 surface composition is usually measured for unsupported, ligand-protected, Ru NPs.^57^ Yet, in a previous DFT-*ab initio* thermodynamics study carried out on the same Ru_55_ model without any surface ligands, 1.6:1 was found to be the optimal hydride-to-surface Ru atom ratio at RT and up to 350 K for a pressure of 1 bar of H_2_.^58^ This ratio might be more representative of a supported Ru NP, where only a rather small part of the surface is stabilized, although it is by a polycyclic support that strongly binds it (Figure 2). In Figure 4a, all 53 or 70 hydrides are accounted for, leading to the calculation of what has been defined as log⁡j^0(Rmax)® . In Figure 4b, only hydrides at distances below 3 Å from the support surface (shown in yellow in Figure 2) are considered, thus yielding what has been defined as log⁡j^0(3)® . For the latter, they represent 12 to 15% of the total number of hydrides, and they are bound to Ru atoms under the direct influence of the support, *i*.*e*., Ru atoms that belong to the cpp1 layer. Thus, these are bridging H atoms between the cpp1 and cpp2 layers.

**Figure 4:**
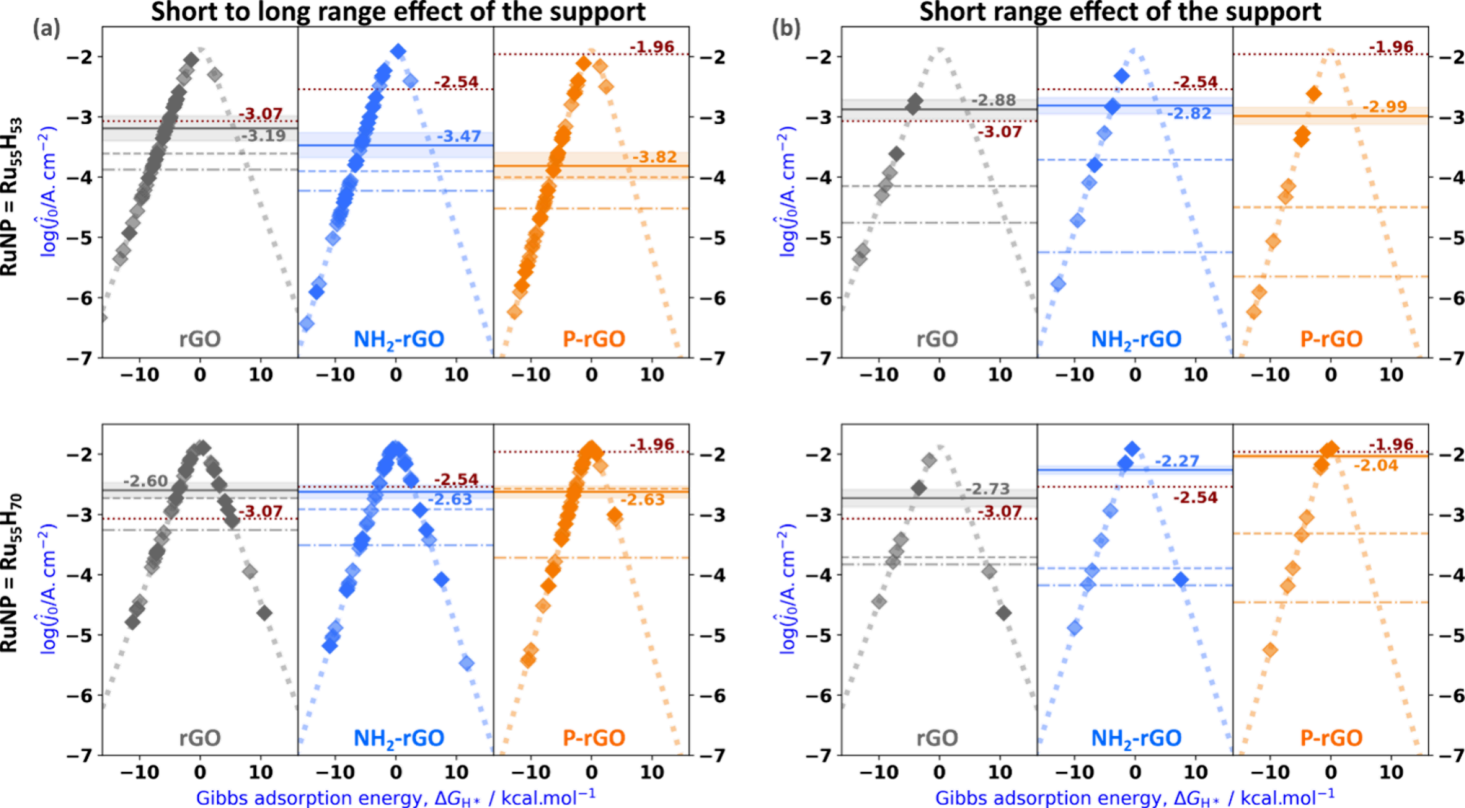
Effect of the support on log⁡j^0(RI)® and on log *ĵ*_0_ calculated in each [*R*_*i*_, *R*_*i*+1_] interval: (a) short to long-range effect (*R* ≤ *R*_max_, see SI, Figure S19); (b) short-range effect (*R* ≤ 3 Å). Calculations done on the Ru_55_H_53_ and Ru_55_H_70_ models are reported on the upper or lower part of each subfigure, respectively. The slope of each volcano depends on the experimentally measured α value. This figure shares some conventions with SI, Figure S20: the experimental log *j*_0_ value is reported in as a horizontal dotted claret line; dark color diamonds, log *ĵ*_0_ values calculated for the supported Ru_55_H_*n*_ model in each [*R*_*i*_, *R*_*i*+1_] interval, between R_0_ = 0 and R*_I_* = *R*_max_ (a) or R*_I_* = 3 Å (b); light color diamonds, log *ĵ*_0_ values calculated for the same intervals, but on the unsupported Ru_55_H_*n*_ model; the corresponding log⁡j^0(RI)® averaged value is also reported in both cases, just above or below horizontal gray, blue, or orange plain lines; dashed lines show results obtained for the unsupported Ru_55_H_*n*_ model in the same geometry as in the supported system, *i.e*., with no hydrides in the grafting domain of the NP (labeled “Ru_55_H_*n*_@X-rGO after support system” in SI, Figure S20); dash–dotted lines show log⁡j^0(RI)® calculated on the original Ru_55_H_*n*_ models developed in ref 58, *i.e*., with hydrides distributed on the whole surface of the Ru_55_ model (labeled “Ru_55_H_*n*_ eq” in SI, Figure S20).

Before comparing the theoretical outcome with the experimental data, a few general characteristics that could be responsible for the differences in the calculated exchange current densities will be reviewed:

#### 4.3.1 Effect of the Increase of the Number of Surface Hydrides Per Unit Area on *j*_0_

As explained in section 2.3 of the SI and in Figure S20, the dash–dotted horizontal lines in Figure 4 correspond to the predicted log *ĵ*_0_ exchange current densities calculated for the hydride sites distributed on the whole Ru surface on unsupported Ru NPs, while the dashed horizontal lines refer to the Ru_55_H_*n*_ model after being supported onto the different x-rGO. It differs from the previous Ru_55_H_*n*_ model in the reduced surface area occupied by the hydrides, since the lower part of the NP interacts with the support. It can be almost systematically observed that in this case, the H---H repulsive interaction weakens their adsorption energy, resulting in an increase of the exchange current density. Provided that rGO surfaces do not involve a significant change in the Ru NP surface composition, the increased number of surface hydrides per unit area could explain in part the differences in electrocatalytic activity between supported and unsupported Ru NPs.

#### 4.3.2 The Support Has a Short-Range Influence Only

Given the volcano plots determined for log⁡j^0(Rmax)® in Figure 4a, the effect of the support is quite small when compared to the unsupported models. There is a moderate increase in the exchange current density, whereas it is much greater when we inspect the short-range effect (below 3 Å) of the graphene surfaces in Figure 4b. This can be understood by examining the diamond symbols reported on the volcano plots, where the dark colors correspond to the hydrides belonging to supported Ru NPs, whereas the light colors correspond to hydrides belonging to unsupported Ru NPs. Most of the dark color diamonds lie quite close to the top in Figure 4b, while most of the light diamonds lie further away from the top. Thus, the adsorption strength of hydrides close to the support is in general small to moderate. In contrast, Figure 4a reveals that most of the hydrides lay on the slopes of the volcanos, both for the supported and the unsupported cases, with no significant differences among them. Thus, they are strongly adsorbed (Δ*G*_H*_ < −5 kcal·mol^–1^), especially when considering the Ru_55_H_53_ model (Figure 4a, top). Consequently, log⁡j^0(3)® is usually less negative than log⁡j^0(Rmax)® , with the only exception of Ru_55_H_70_@rGO, for which the two predicted values are very close (−2.73 vs. −2.60). In other words, the support acts locally as a macrocyclic ligand which increases the HER catalytic activity of its neighboring Ru atoms only by decreasing the Δ*G*_H*_ values.

#### 4.3.3 A Synergy of Effects

If we now focus on Figure 4b, in order to discuss the intrinsic effect of the surface on Δ*G*_H*_ and by consequence on log *ĵ*_0_, this effect, combined with the H---H repulsive interaction discussed in (i), tends to bring log *ĵ*_0_ close to the top of the volcano (∼ −2), a criterion of strong HER activity for the corresponding catalysts (Ru_55_H_53_@X: −2.88, −2.82, −2.99; Ru_55_H_70_@X: −2.73, −2.27, −2.04, with X = rGO, NH_2_-rGO, P-rGO, respectively). The intrinsic effect of the support is strong when compared with the unsupported Ru_55_H*_n_* models, with an increase of log⁡j^0(3)® for Ru_55_H_53_@X of +1.27, +0.90, +1.51, as well as for Ru_55_H_70_@X of +0.98, +1.64, +1.28 (these are the differences between plain lines and dashed lines in Figure 4b, with X = rGO, NH_2_-rGO, and P-rGO, respectively).

Finally, we will determine which model best agrees with the experimental measurements. If we now go back to the experimental HER activity of the three studied materials, it decreases in the order **Ru-r@P-rGO** (log *ĵ*_0_: −1.96) > **Ru-r@NH**_**2**_ **-rGO** (log *ĵ*_0_: −2.54) > **Ru-r@rGO** (log *ĵ*_0_: −3.07) (horizontal dotted claret lines in Figure 4). Can the DFT-based simulations explain this trend? If we analyze the overall effect of the support on log *j*_0_ given by the calculation of log⁡j^0(Rmax)® (Figure 4a) and compare it with the experimentally measured values, we can see in that log⁡j^0(Rmax)® does not follow the experimental trend for the grafted Ru_55_H_53_ model. A fair agreement is observed between theory and experiment for **Ru-r@rGO** (−3.19 vs. −3.07), but there is a strong disagreement for **Ru-r@NH**_**2**_ **-rGO** (−3.47 vs. −2.54) and an even worse discrepancy for **Ru-r@P-rGO** (−3.82 vs. −1.96). Concerning the Ru_55_H_70_ model, the predicted log⁡j^0(Rmax)® values are in better agreement, although the experimental trend is not obtained since the exchange current is predicted to be almost the same between the three Ru_55_H_70_ supported models ( $\log{ĵ}_{0}$ : −2.60, −2.63, and −2.63), in contrast with the progressive increase of the exchange current density observed in the experiments between **Ru-r@rGO**, **Ru-r@NH**_**2**_ **-rGO**, and **Ru-r@P-rGO**, respectively. Thus, the results plotted in Figure 4a do not follow the experimental trend nor the expectations from the electronic structure analysis, particularly the possible higher activity of **Ru-r@P-rGO** according to its d-band center value. This is just an apparent contradiction, given that all the Ru NPs surface site activities are averaged in log⁡j^0(Rmax)® , even those that are opposite to the support, and therefore not sensitive to the effect of the graphene-derived support. However, this is not the case for the 12 to 15% of hydrides accounted for in the calculation of log⁡j^0(3)® (Figure 4b). With −2.88, the predicted value is reasonably close to the experimental value (−3.07) for Ru_55_H_53_@rGO. The agreement is less convincing for the other materials, with a moderate underestimation for Ru_55_H_53_@NH_2_-rGO (−2.82 instead of −2.54) and a strong underestimation for Ru_55_H_53_@P-rGO (−2.99 instead of −1.96). The resulting computed trend, Ru_55_H_53_@P-rGO < Ru_55_H_53_@rGO < Ru_55_H_53_@NH_2_-rGO, is not in line with the experiments. In contrast, the experimental trend is achieved by the Ru_55_H_70_ model. Although log⁡j^0(3)® is a bit overestimated for Ru_55_H_70_@rGO (−2.73 vs. −3.07) and Ru_55_H_70_@NH_2_-rGO (−2.27 vs. −2.54) and slightly underestimated for Ru_55_H_70_@P-rGO (−2.04 vs. −1.96), the predicted values follow the very same experimental relative activity values trend, the best catalyst for HER being Ru_55_H_70_@P-rGO, followed by Ru_55_H_70_@NH_2_-rGO and finally Ru_55_H_70_@rGO. Given the wealth of experimental and theoretical knowledge we have on small Ru NPs,^58^ it is reasonable to consider that their surface can accommodate at least 1.5 H^–^ per surface ruthenium atom when stabilized by a single macrocycle-like ligand. To complement the theoretical analysis, detailed insights into the electronic structure of the Ru_55_H_70_@X systems are presented in the SI (Figure S21). Notably, these findings confirm the intrinsic stability of the Ru_55_ core. Additionally, they provide further energy-related evidence regarding the coordination strength between the Ru NP model and the support. The results highlight both a strong Ru–P bond interaction energy index and increased activity of the cpp1 layer of Ru_55_H_70_@P-rGO, particularly in relation to its d-band center.

## 5 Conclusions

In this work, we have studied the effect of N- and P-doping of rGO onto the electrocatalytic activity of the respective supported Ru NPs, observing an outstanding activity (η_10_ of only 2 mV) for the P-doped material. Owing to a thorough modeling of the supports and an accurate DFT analysis of the rGO-supported Ru NPs, a consistent trend between computed and experimental exchange current densities have been obtained. This has involved being accurate enough to reproduce small variations of the average hydrogen adsorption free energy descriptor, Δ*G*_H*_. Furthermore, theoretical DFT calculations have shown to be an alternative and reliable way to estimate the amount of surface hydrides in Ru NPs when supported onto heterogeneous surfaces such as doped-rGO, conditions where the experimental titration of hydrides by reaction with 2-norbornene^59^ is not practical nor feasible. A good agreement between theory and experiments has been found with a Ru_55_H_70_ model, *i*.*e*., a model containing 1.6 H^–^/Ru_surf_, but only on the assumption that the observed experimental activity of Ru NPs arises solely from the Ru atoms close to the graphene-derived support (below 3 Å), which are generally the more active ones. Moreover, in this scenario we should not discard the possibility that some Ru NPs may be in contact with the support through more than one ccp layer, that is, that some NPs were somehow embedded in the carbonaceous support. This would increase the number of high activity sites and thus the overall HER electrochemical capacity of the whole material. Unfortunately, it is not in our hands to experimentally determine the real number of ccps that are in contact with the rGO surfaces. In any case, the resulting calculated exchange current density, *j*_*0*_, which is a measure of the efficiency of the catalyst with respect to HER, seems to be related to a combination of several parameters: the high d-band center of Ru atoms coordinated to the substrate, the weaker charge transfer between Ru NPs and the support, and a sufficiently high hydride coverage that slightly destabilizes surface hydrides through increased H---H repulsions.

In this work, both the experimental and theoretical results converge and confirm the positive synergistic effect between the heteroatoms (especially P) and the Ru NPs to obtain excellent HER electrocatalytic activities. This work paves the way for a better understanding of the intricate interrelated factors determining the HER electrocatalytic activity of supported Ru NPs in heteroatom-doped rGO surfaces, thus opening new avenues for improved designs of superior HER electrocatalysis.

## Supplementary Material



## Notes

Microscopy images, Raman data, XPS data, NMR data, LSV data, bulk electrolysis data, electrochemical parameters determination, relevant related literature data, and DFT computational details (PDF)
