# Supplementary material for: Effect of Nitrogen and Phosphorus Doping of Reduced Graphene Oxide in the Hydrogen Evolution Catalytic Activity of Supported Ru Nanoparticles
Source: ACS Appl Mater Interfaces. 2025 Jan 20;17(4):6198–210. doi: 10.1021/acsami.4c15547 (PMC12131230; doi:10.1021/acsami.4c15547)
Supplement: Supplementary file 1 [file am4c15547_si_001.pdf]

SUPPORTING INFORMATION

Effect of Nitrogen and Phosphorus Doping of Reduced Graphene Oxide in the Hydrogen Evolution Catalytic Activity of Supported Ru Nanoparticles

Laura Mallón,[‡♣] Javier Navarro-Ruiz,[‡♦] Christian Cerezo-Navarrete,[‡♥] Nuria Romero,[♠] Iker del Rosal,[♦] Jordi García-Antón,[♣] Roger Bofill,[*♣] Luis Miguel Martínez-Prieto,[*♥,•] Karine Philippot,[*♠] Romuald Poteau[*♦] and Xavier Sala [*♣]

♣ Departament de Química, Universitat Autònoma de Barcelona, 08193 Bellaterra (Catalonia), Spain. E-mail: Roger.Bofill@uab.cat; Xavier.Sala@uab.cat

♦ Université de Toulouse; INSA, UPS, CNRS; LPCNO (IRSAMC), 135 avenue de Rangueil, F-31077 Toulouse, France. E-mail: romuald.poteau@insa-toulouse.fr

♥ ITQ, Instituto de Tecnología Química (CSIC-Universitat Politècnica de València), Av. de los Naranjos S/N 46022, Valencia, Spain. E-mail: luismiguel.martinez@csic.es

♠ CNRS, LCC (Laboratoire de Chimie de Coordination), UPR8241, Université de Toulouse, UPS, INPT, Toulouse cedex 4 F-31077, France. E-mail : Karine.Philippot@lcc-toulouse.fr

• IIQ, Instituto de Investigaciones Químicas (CSIC-Universidad de Sevilla), Avda. Américo Vespucio 49, 41092 Seville, Spain.

[‡] All these authors have contributed equally to this work.

Table of Contents

Page

1. Figures and Tables

TEM and HRTEM images of **Ru@X-rGO** (X = none, NH_2 or P) 3

Raman spectra of P-rGO and **Ru@P-rGO** 4

XPS spectra of X-rGO and **Ru@X-rGO** (X = none, NH_2 or P) 5

Quantification of Ru(0) and RuO_2 by XPS of **Ru@X-rGO** (X = none, NH_2 or P) before and after chemical reduction (Table S1). 6

^{31}P MAS NMR spectrum of P-rGO 7

TEM images of **Ru@X-rGO** (X = none, NH_2 or P) after catalysis 7

LSVs for **Ru-r@X-rGO** (X = none, NH_2 or P) before and after 12 h-CP 8

H_2-monitored bulk electrolysis of **Ru@X-rGO** (X = none, NH_2 or P) 8

α determination for **Ru-r@X-rGO** (X = none, NH_2 or P) 9

C_{DL} determination for **Ru@X-rGO** (X = none, NH_2 or P) 10

Comparison of most relevant G-based and Ru/G-based HER nanocatalysts at acidic pH (Table S2) 11

2. DFT computational details and additional analysis

2.1. Design of the structural models 13

2.1.1. Design of the supports 13

2.1.2. Grafting of Ru_{55} NPs on G-derived supports 17

2.2. Volcano plots and DFT calculations of H adsorption energies 19

2.3. Evaluation of the range effect of the supports on H adsorption energies 23

2.4**.** DOS and pCOHP analysis of the support - Ru-r interactions in $Ru_{55}H_{70}$ 25

3. References 26

1. Figures and Tables

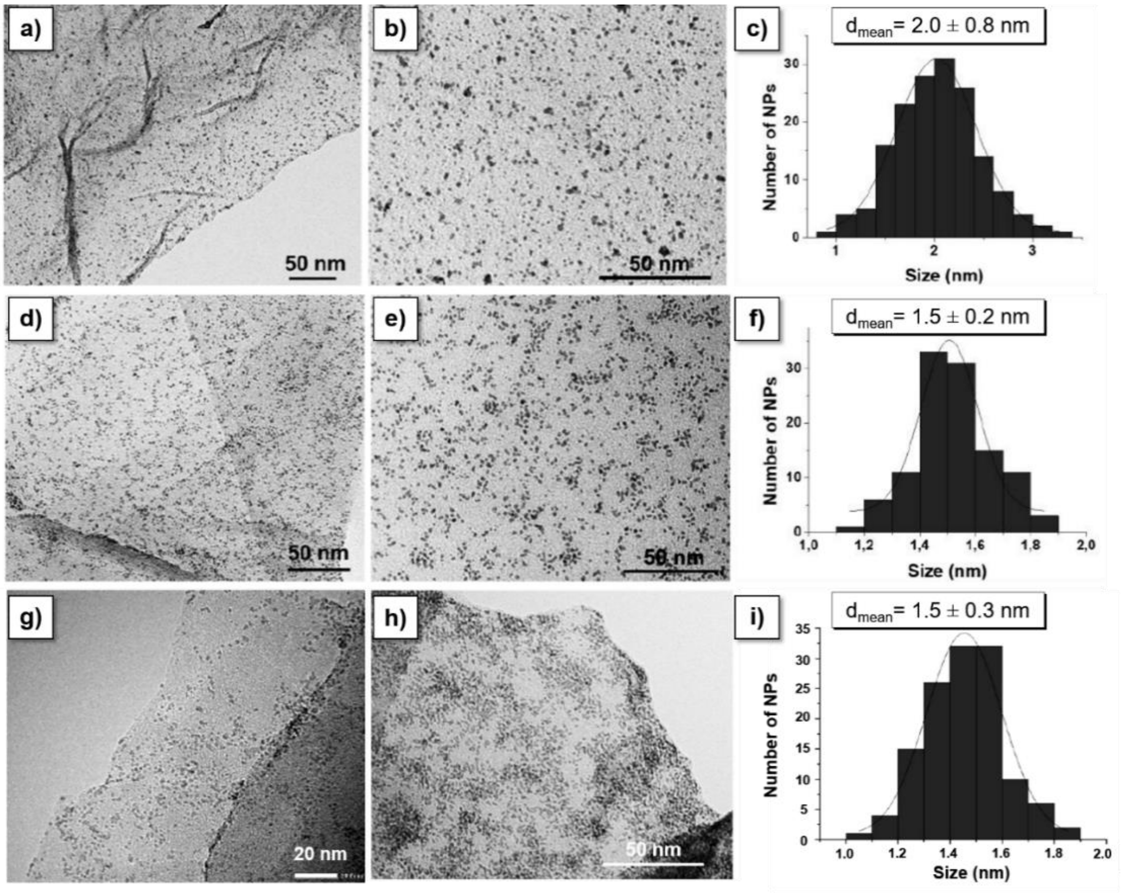

Figure S1. TEM images and corresponding size distribution histograms of (a-c) **Ru@rGO**, (d-f) **Ru@NH$_2$-rGO** and (g-i) **Ru@P-rGO**. Figures 1a-f are reproduced with permission from ref. 1. Copyright 2019 Elsevier Inc.

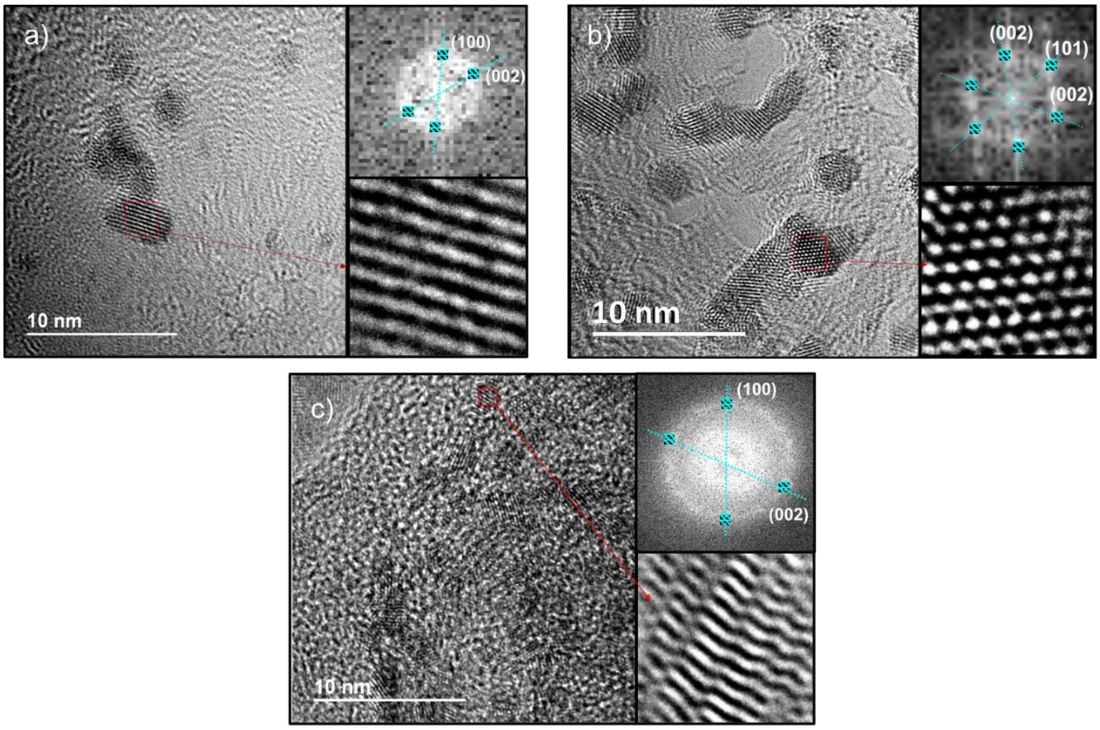

Figure S2. HRTEM micrographs of (a) **Ru@rGO**, (b) **Ru@NH$_2$-rGO** and (c) **Ru@P-rGO**. HRTEM image of **Ru@P-rGO** (left, right bottom) and Fourier Transform analysis with the planar reflections (right, top), which displays reflections to the (100) and (002) atomic planes with a lattice fringe spacing of 2.289 Å and 2.165 Å, respectively. Figure S2b is reproduced with permission from ref. 1. Copyright 2019 Elsevier Inc.

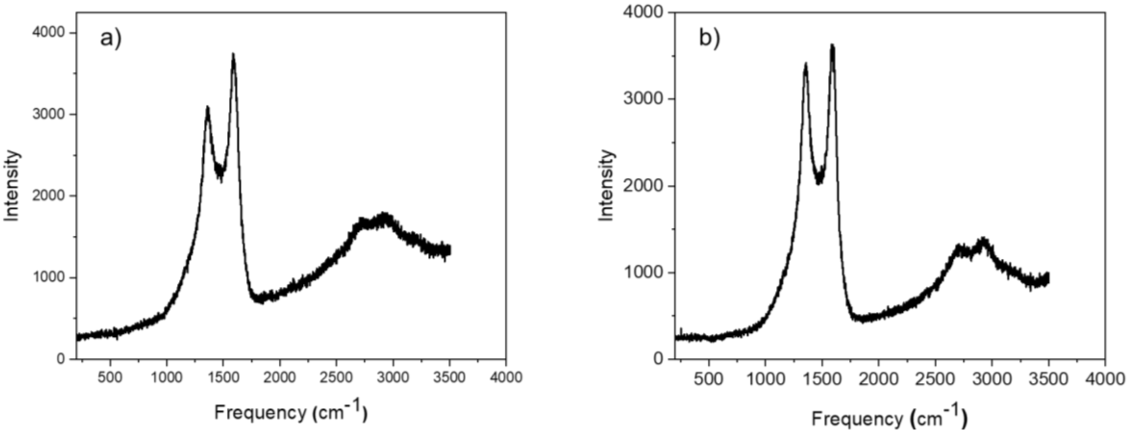

Figure S3. Raman spectra of (a) P-rGO and (b) **Ru@P-rGO**.

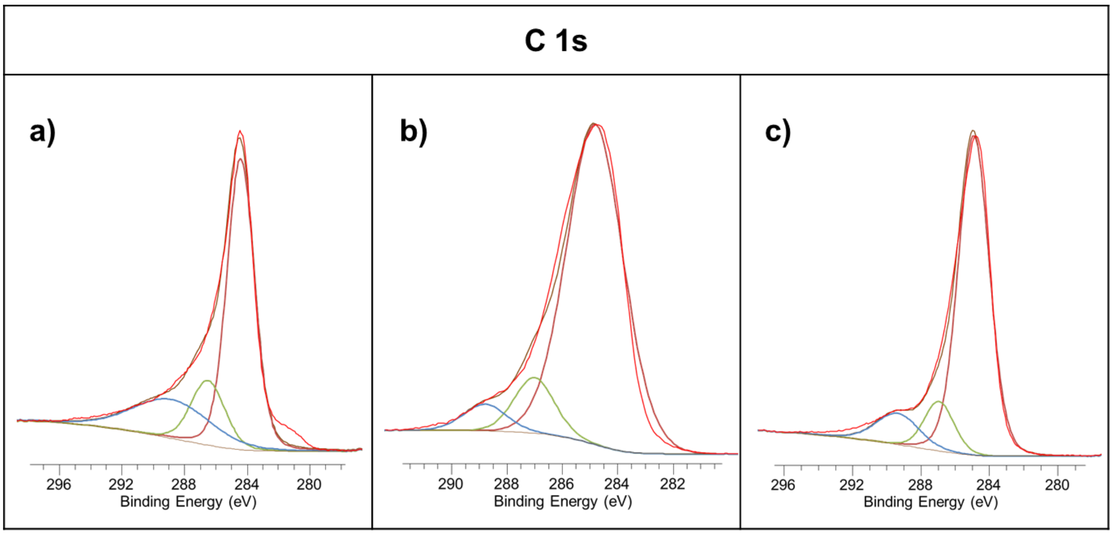

Figure S4. XPS spectra of C 1s signal of (a) rGO, (b) NH_2-rGO and (c) P-rGO. Figure S4b is reproduced with permission from ref. 1. Copyright 2019 Elsevier Inc.

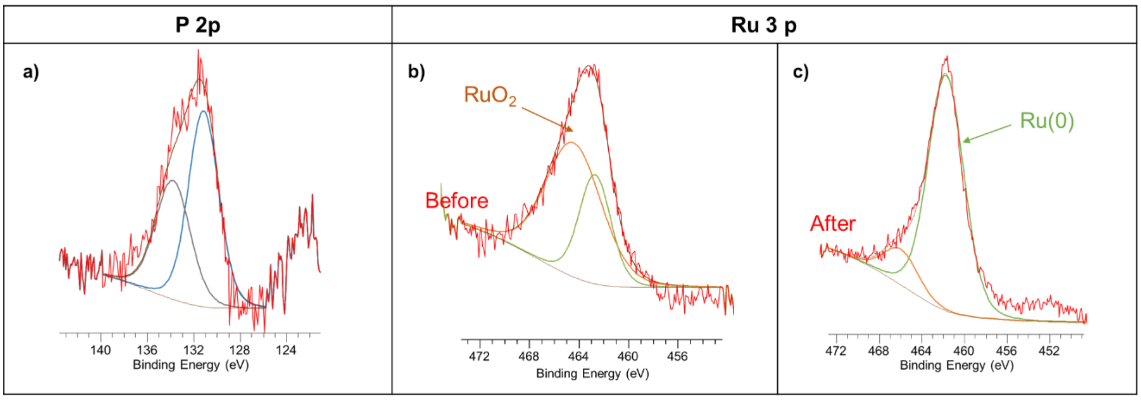

Figure S5. XPS spectra of (a) P 2p signal of P-rGO, and the Ru 3p signal of **Ru@P-rGO** (b) before and (c) after reduction conditions (180 °C under H_2 atmosphere for 5 h).

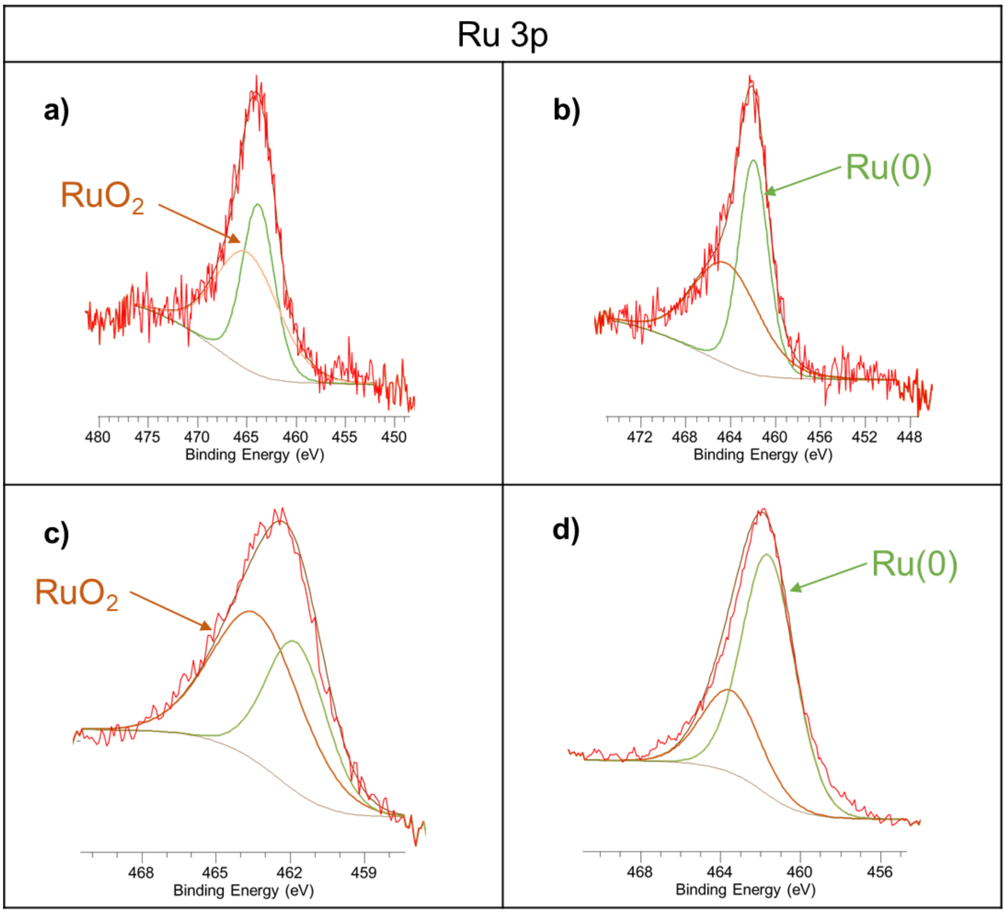

Figure S6. XPS spectra of Ru 3p signal of **Ru@rGO** (a) before and (b) after reduction (180 °C under H_2 atmosphere for 5 h), and **Ru@NH$_2$-rGO** (c) before and (d) after reduction conditions (180 °C under H_2 atmosphere for 5 h). Figures S6c-d are reproduced with permission from ref. 1. Copyright 2019 Elsevier Inc.

Table S1. Quantification of Ru(0) and RuO_2 by XPS of **Ru@rGO**, **Ru@NH$_2$-rGO** and **Ru@P-rGO** before (left) and after (right) reduction (180 °C under H_2 atmosphere for 5 h).

Material	Before reduction (%)		After reduction (%)	
	Ru(0)	RuO_2	Ru(0)	RuO_2
**Ru@rGO**	42.6	57.4	48.4	51.6
**Ru@NH$_2$-rGO**	40.7	59.3	72.6	27.4
**Ru@P-rGO**	31.6	68.3	87.3	12.6

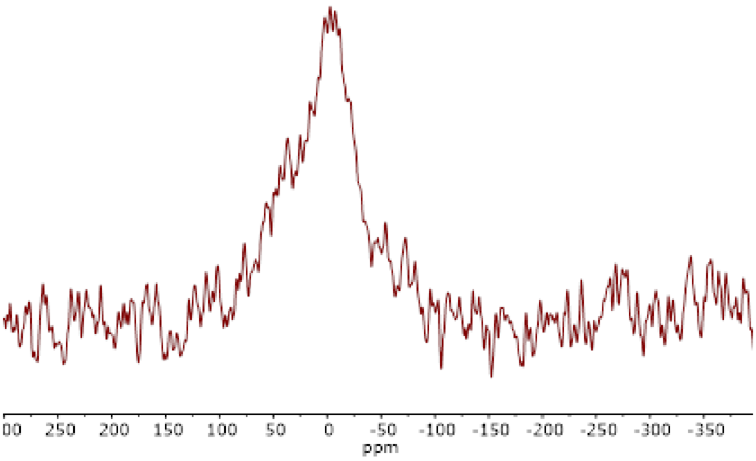

Figure S7. ^{31}P MAS NMR spectrum of P-rGO.

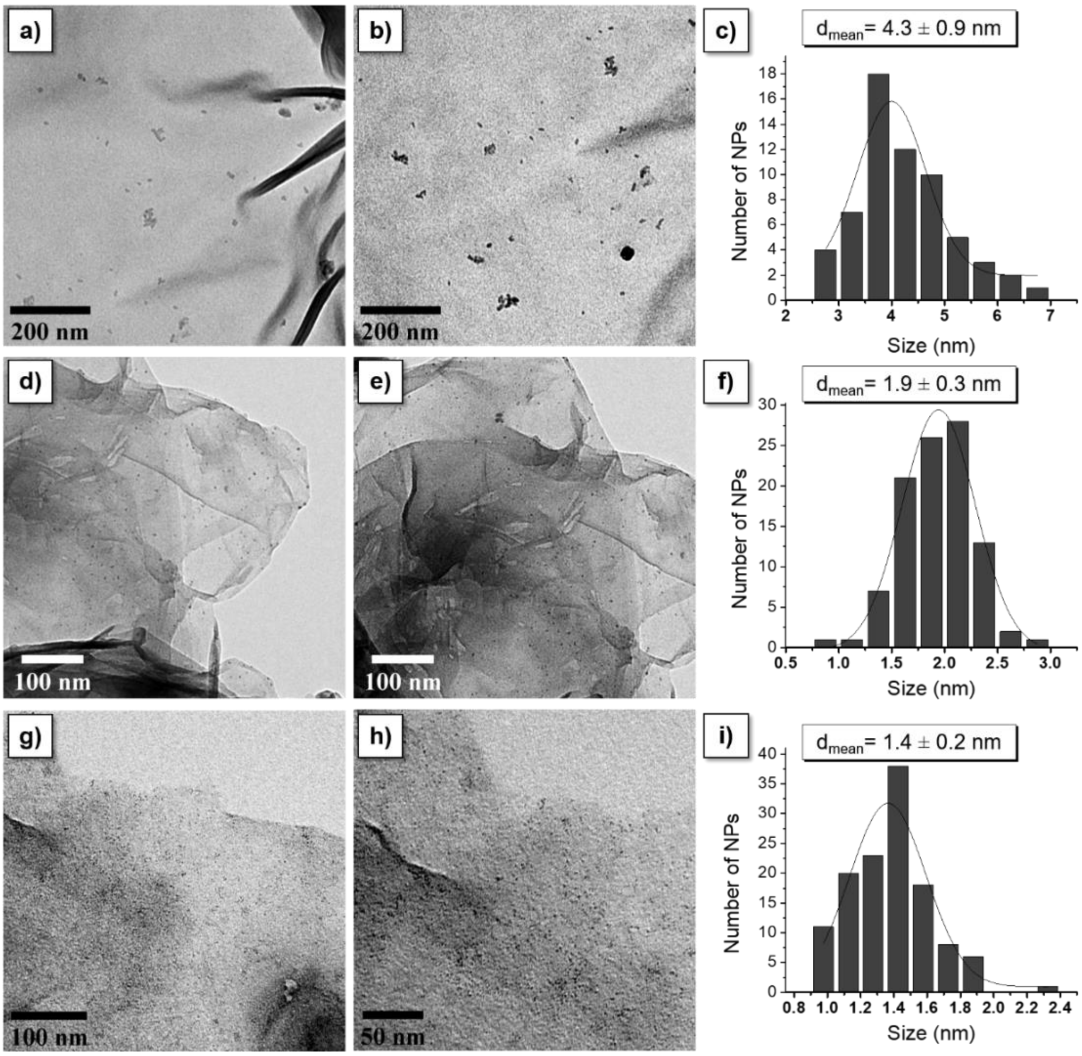

Figure S8. TEM images and corresponding size histograms of (a-c) **Ru@rGO**, (d-f) **Ru@NH_2-rGO** and (g-i) **Ru@P-rGO** after 2 h under catalytic conditions (j = -10 mA·cm^{-2}).

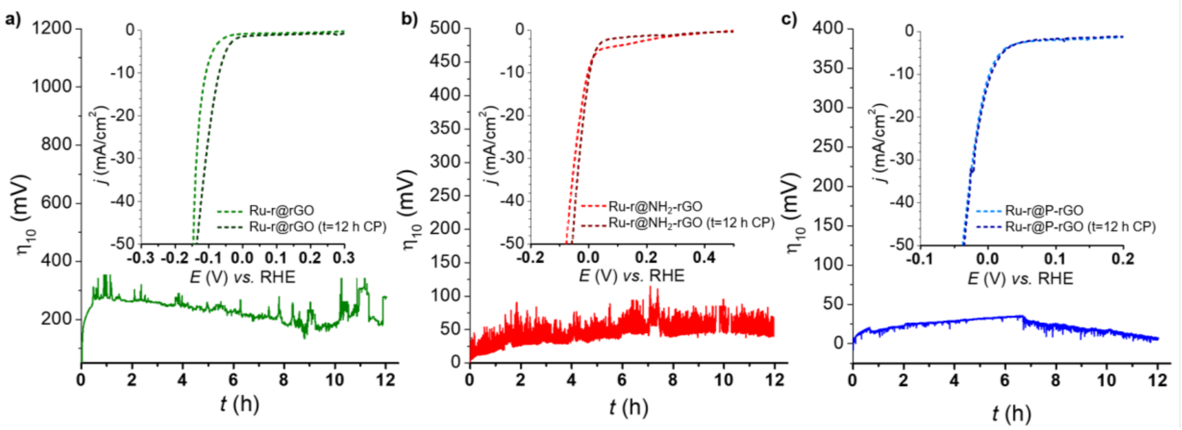

Figure S9. LSV of (a, green) **Ru-r@rGO**, (b, red) **Ru-r@NH$_2$-rGO** and (c, blue) **Ru-r@P-rGO** before (light color) and after (dark color) a 12-h chronopotentiometry experiment at a *j*= -10 mA·cm^{-2}.

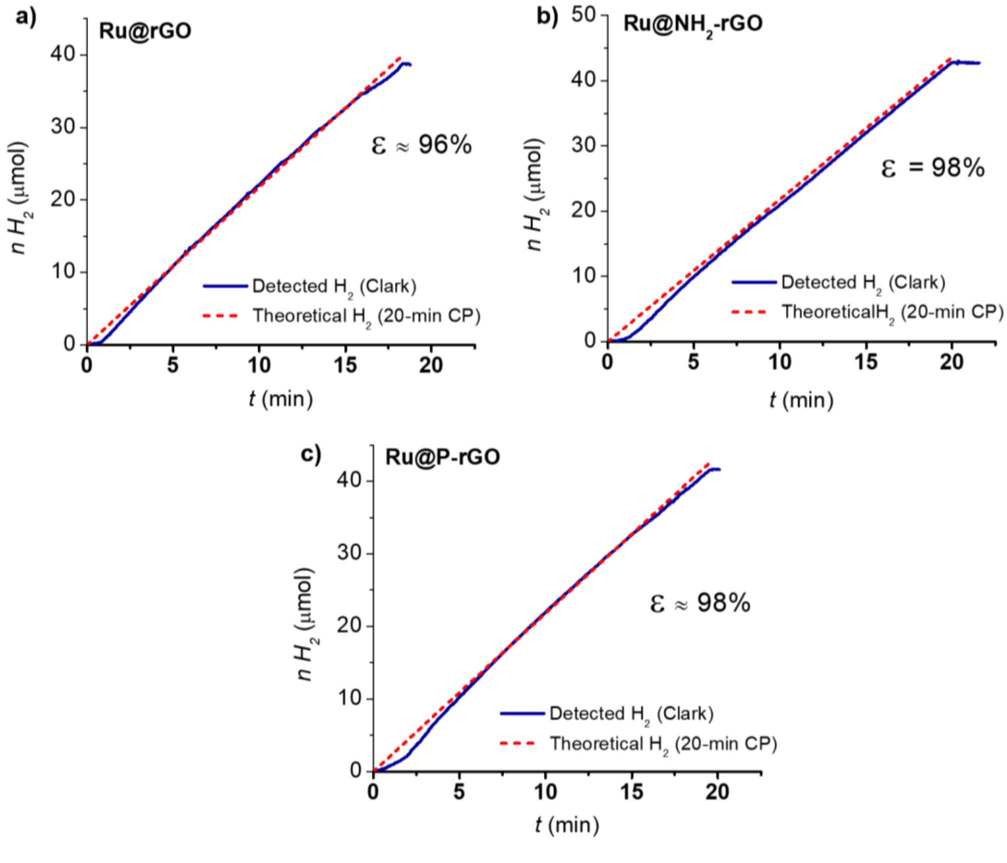

Figure S10. H$_2$-monitored current-controlled bulk electrolysis of (a) **Ru@rGO**, (b) **Ru@NH$_2$-rGO** and (c) **Ru@P-rGO** in 1 M H_2SO_4. The production of H_2 was quantified in the gas phase by using a Clark-type electrode.

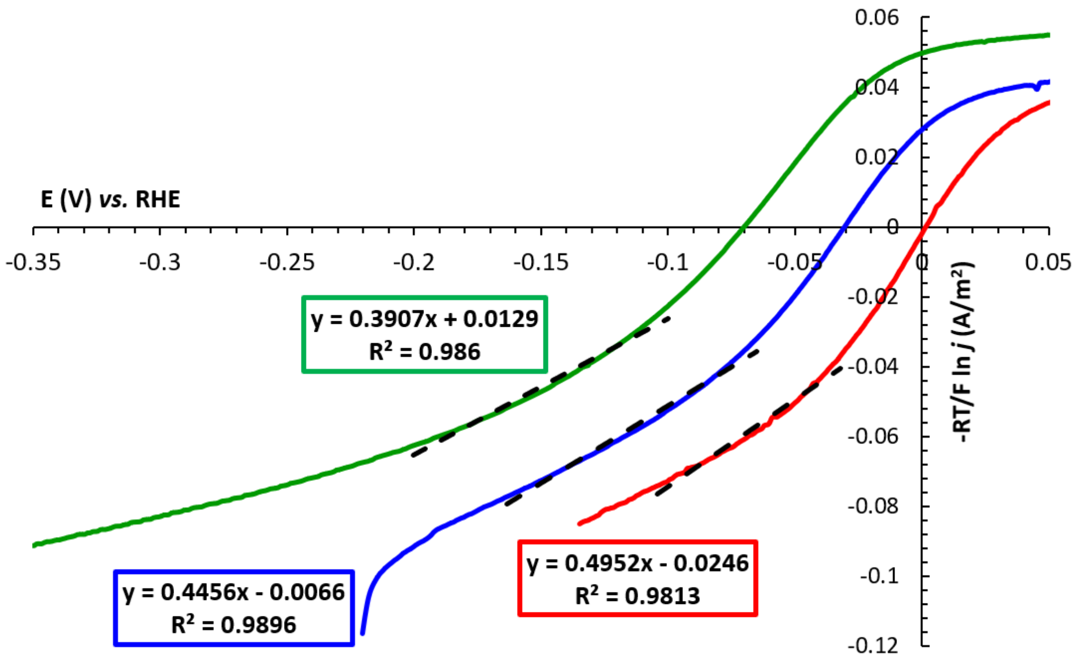

Figure S11. Determination of cathodic transfer coefficient (α) for **Ru-r@rGO** (green), **Ru-r@NH$_2$-rGO** (blue) and **Ru-r@P-rGO** (red).

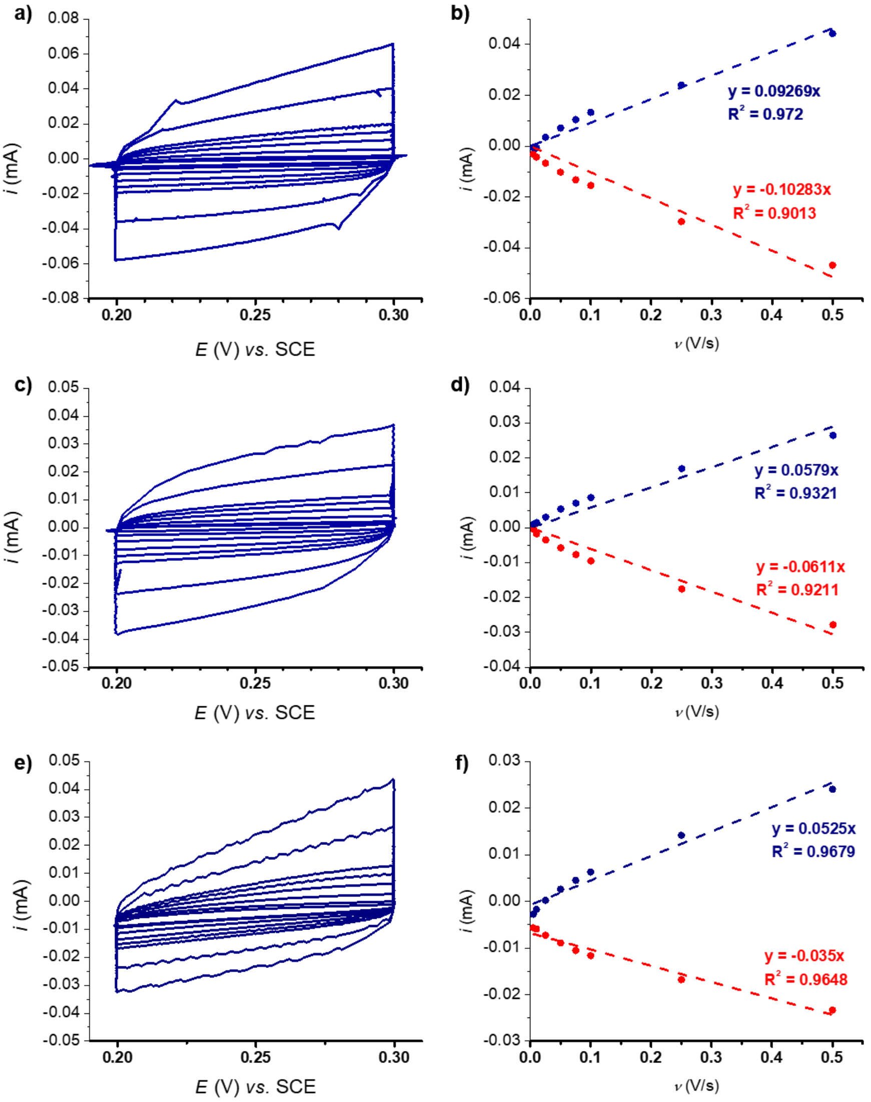

Figure S12. Representative multi-CV experiments at different scan rates for C_{DL} determination of (a) **Ru@rGO**, (c) **Ru@NH$_2$-rGO** and (e) **Ru@P-rGO**. Plot of current values at 0.25 V *vs.* SCE for the different scan rates in 1 M H_2SO_4 for (b) **Ru@rGO**, (d) **Ru@NH$_2$-rGO** and (f) **Ru@P-rGO**.

Table S2. Comparison of the most relevant graphene-derived and Ru/graphene-based HER nanoelectrocatalysts with our materials under acidic conditions. Commercial Pt/C and Ru@C electrodes have also been added for the sake of comparison. Parameters: mean diameter (Ø), onset overpotential (η_0, mV), overpotential at $|j|$ =10 mA·cm^{-2} (η_{10}, mV), Tafel slope (b, mV·dec^{-1}) and exchange current density ($|j_0|$, mA·cm^{-2}).

Entry	Catalyst	Ø (nm)	η_0 (mV)	η_{10} (mV)	b (mV·dec^{-1})	$\lvert j_0 \rvert$ (mA·cm^{-2})	Ref.
1	**Ru-r@rGO**	2.0	0	71	65	0.85	This work
2	**Ru-r@NH_2-rGO**	1.5	≈ 0	30	56	2.87	This work
3	**Ru-r@P-rGO**	1.5	≈ 0	2	51	10.88	This work
4	Ru/G-r	1.9	≈ 0	29	48	2.50	2
5	Ru/P-G-r	1.5	≈ 0	15	49	4.97	1
6	GCE-S-GNs-1000-CB-Ru	30	≈ 60	80	61 (Tafel)<br>71 (EIS)	0.541<br>0.431	3
7	Ru-GLC	2-5	3	35	46	-	4
8	Ru_2P/RGO	<7	≈ 0	22	29	2.2	5
9	Ru_2P@PNC	18	≈ 0	15	28	-	6
10	Ru@GnP	2	≈ 0	13	30	-	7
11	N-G	-	≈ 250	490	116	-	8
12	P-G	-	≈ 300	550	133	-	8
13	N,P-G	-	≈ 240	420	91	0.00024	8
14	N,B-CN	-	≈ 410	710	198	-	9
15	N-CN	-	≈ 400	620	159	-	9
16	N,P-CN	-	≈ 340	550	139	-	9
17	N,S-CN	-	≈ 100	290	77	-	9
18	N-G	-	499	-	405	86	10
19	N,P-G	-	399	-	565	265	10
20	P-G	-	536	-	348	76	10
21	P,N-G	-	247	380	126	21	10
22	VG	-	≈ 375	-	158	-	11
23	N-VG	-	≈ 200	290	121	-	11
24	Ru/NG-750	3-7	≈ 0	53	44	-	12
25	Ru@CN	2.37	≈ 70	126	-	-	13
26	Ru-NGA	3.5	≈ 15	55	32	-	14
27	Ru@NC	2.1	≈ 10	62	40	-	15
28	Commercial Ru-b	-	70	150	65	0.14	16
29	Commercial Pt/C	-	0	27	32	1.4	16

Legend: Ru/G-r, Ru NPs supported on alginate-derived graphene after a reductive treatment; Ru/P-G-r, Ru NPs supported on alginate-derived P-doped graphene after a reductive treatment; GCE-S-GNs-1000, glassy carbon modified sulphur-doped graphene nanosheets heat treated at 1000 °C; GLC, graphene-layered carbon; RGO, reduced graphene oxide; PNC, P,N-doped C nanofibers; GnP, graphene nanoplatelets; N-G, N-doped graphene; P-G, P-doped graphene; N,P-G, N,P-doped graphene; N,B-CN, N,B-doped carbon nanosheets; N-CN, N-doped carbon nanosheets; N,P-CN, N,P-doped carbon nanosheets;

N,S-CN, N,S-doped carbon nanosheets; P,N-G, P,N-doped graphene; VG, vertical graphene; N-VG, N-doped vertical graphene; Ru/NG-750, Ru nanoclusters on N-doped graphene prepared at 750 °C; Ru@CN, Ru NPs over N-doped carbon; Ru-NGA, Ru-modified N-doped graphene aerogel, Ru@NC, Ru NPs embedded in N-doped carbon; Ru-b: Ru-black.

2. DFT computational details

2.1. Design of the structural models

This subsection is organized as follows. Firstly, results based on experimental data are shown, related to the computational modelling of the rGO, NH_2–rGO, and P–rGO supports from pristine graphene. Then, the anchoring of the hydrogenated ruthenium nanoparticle (Ru NP) is discussed, paying special attention to the metal–support interaction and structural changes derived from the coordination of the metallic system on the different carbon materials.

2.1.1. Design of the supports. The first step in the development of supported metal catalysts is the design of the supports. For this, graphene was used as a model carbon support, given its excellent properties in supported catalysis.[17] Lattice imperfections are inevitably introduced during its growth or processing. SW defects, single and multiple vacancies (SVs and MVs), carbon, foreign adatoms, and substitutional impurities are typical stable point defects that have been predicted and experimentally observed. Among these, the V_2(555−777) defect, consisting of a double vacancy reconstructed with three pentagonal and three heptagonal cells of carbon atoms in the graphene lattice, appears as one of the most favored and commonly observed vacancy defects in electron microscopy analyses.[18] Therefore, the introduction of these V_2(555−777) defects was first considered on pristine graphene, causing no deformation in the surface plane.

The next step was the functionalization of the carbon surface. From the elemental analyses and XPS data previously reported for rGO and NH_2–rGO,[1] as well as those shown in Figures S4, S5 and S7 for P–rGO, the molar ratio and density of functional groups, respectively, could be deduced for each support. In order to consider each of these functional groups detected and quantified experimentally, the modelling of the different moieties was carried out through a sequence of serial optimizations on the defective graphene, similarly to the procedure previously reported in our group.[19] The addition/creation of functional groups is carried out homogeneously, with those found within the carbon lattice being introduced first, followed by increasingly larger groups. The final results can be seen in the right part of Figure S13. In the case of rGO, the order was: carboxyl → hydroxyl → epoxide. The moderate concentration of oxygenated groups induces a loss of planarity in the graphitic system, giving rise to the formation of remarkable superficial corrugations. Regarding NH_2–rGO, the order was: graphitic–N + pyridinic–N + pyrrolic–N → carboxyl → amino → hydroxyl → epoxide. The N-doped support presents a higher density of functionalities and, therefore, a higher deformation of the carbon surface. It is interesting to mention the increase in vacancy defects on the carbon lattice due to the presence of pyridine and pyrrole rings, including some protonated ones. Finally, the order in the P–rGO was: graphitic–P + graphitic–(P=O) → carboxyl → hydroxyl. This support presents the least of the functionalization, partly due to the low phosphorus incorporation in the graphene oxide shell. This leads to a higher concentration of pristine graphitic areas and therefore less rippling of the carbon system.

The thermal stability of previously optimized rGO (clean and doped with N and P)-based supports was investigated through AIMD simulations at a temperature of 450 K (according to the reaction conditions). First, the simulation analysis evidenced in all three cases a relatively fast total energy convergence over time, as shown in Figure S14. The supports reached dynamic equilibrium at approximately 5 ps, after which only a constant oscillation of energy is noticeable. Structural changes were detected, especially in rGO and NH_2–rGO. In the former, an entire –

COOH group was released, while in the latter, CO_2 and H_2O were formed as a result of condensation between a carboxyl and a hydroxyl group. P–rGO showed only rather conformational changes derived from out-of-plane vibrations of reduced phosphorus (unlike oxidized phosphorus, P=O). The final geometry of the simulations was relaxed (right part of Figure S13), presenting only small rotations (of carboxyl and amino groups) and deformations (out of plane in the graphitic region).

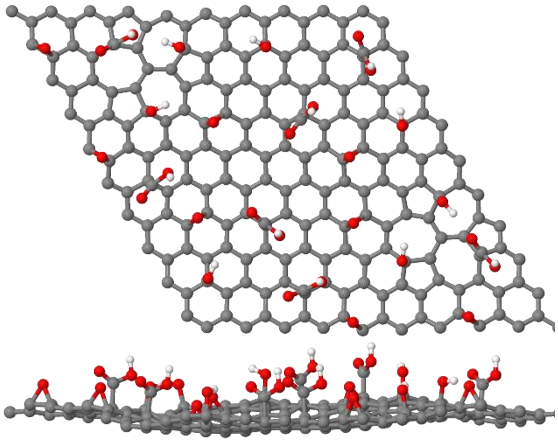

a) rGO (initial)

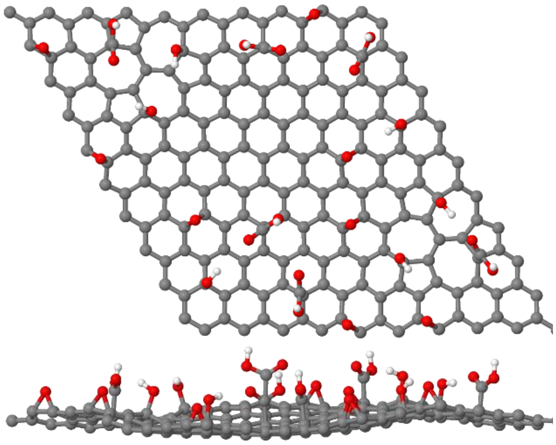

b) rGO (final)

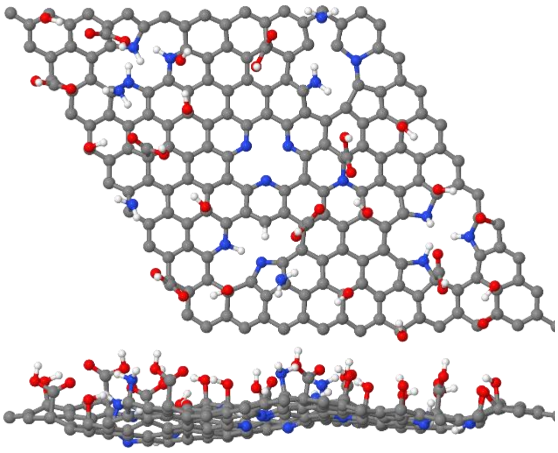

c) NH_2–rGO (initial)

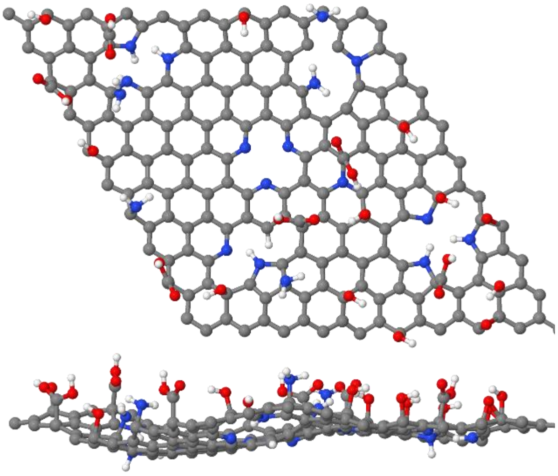

d) NH_2–rGO (final)

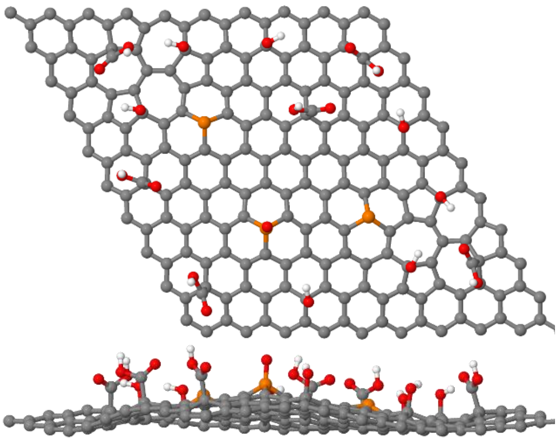

e) P–rGO (initial)

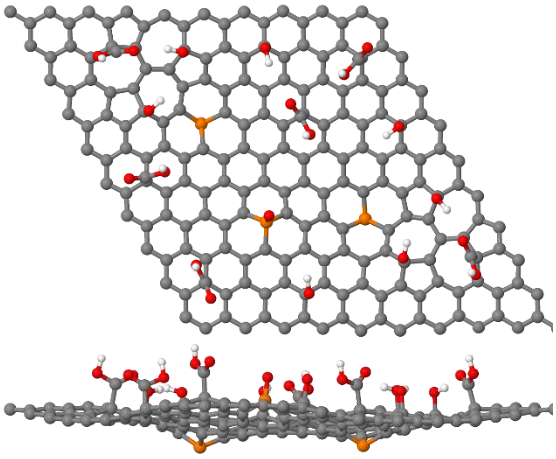

f) P–rGO (final)

Figure S13. Top and side views of the initial and final (**a-b**) rGO, (**c-d**) NH_2–rGO, and (**e-f**) P–rGO model carbon supports. Atomic color code: P (Orange); O (Red); N (Blue); C (Grey); H (White).

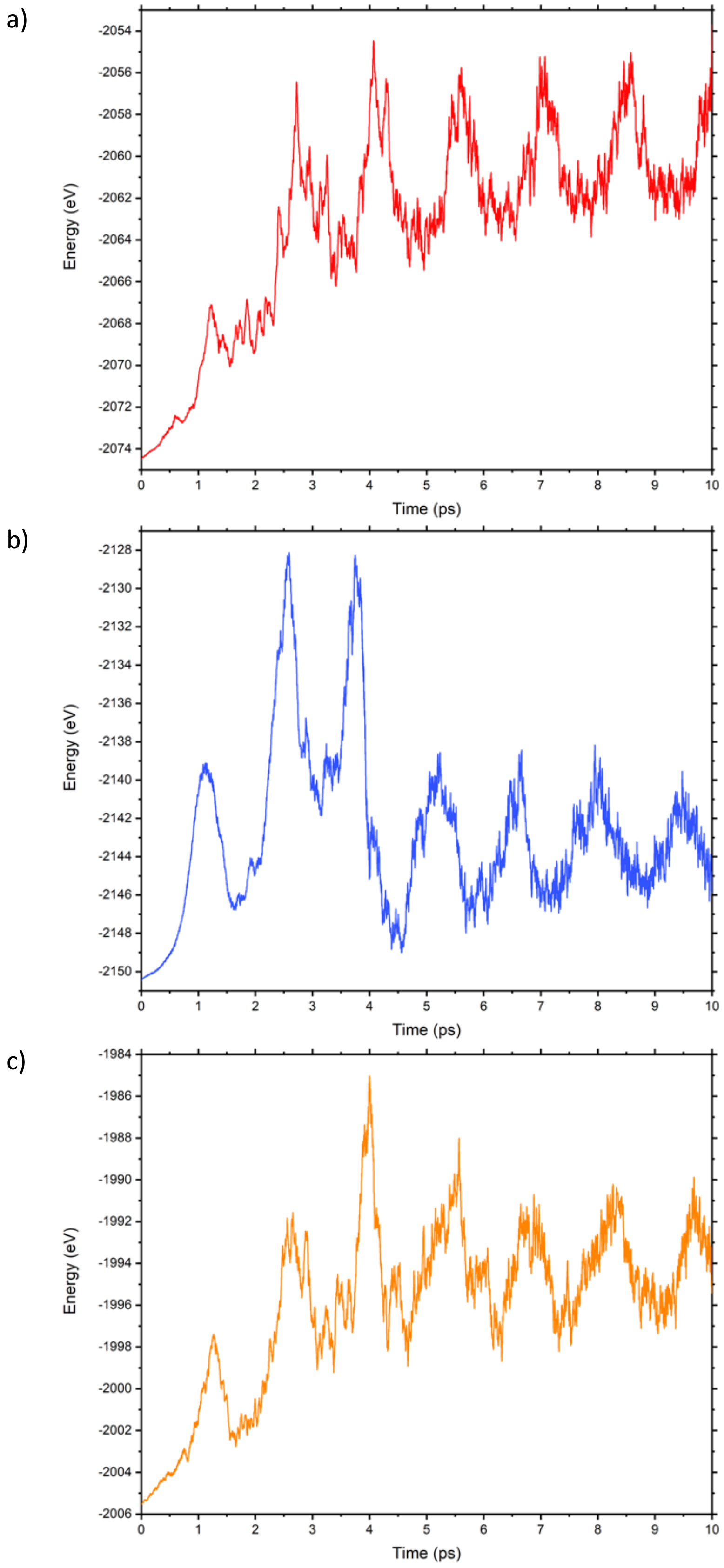

Figure S14. AIMD of the (**a**) rGO, (**b**) NH_2–rGO, and (**c**) P–rGO model carbon supports: total energy as a function of time.

2.1.2. Grafting of Ru_{55} NPs on G-derived supports. Once the pristine graphene has been oxidized into rGO and the corresponding N- and P-doped systems, they can be used as supports for metal deposition. Thus, a hcp-based 55-atoms Ru NP model was deposited on the functionalized surfaces. Such nanoparticles are representative of highly dispersed Ru catalysts (see Figure S1 for the TEM images and size distribution histograms of the **Ru@rGO**, **Ru@NH_2–rGO**, and **Ru@P–rGO** catalysts). Additionally, since the experiments were carried out under H_2 pressure and dihydrogen easily dissociates on the surface of the NP, it is expected to be basically covered with surface hydrides. Consistent with what was previously reported for hydride Ru NPs,[20] a hydrogen-to-surface-metal ratio of approximately 1.2:1 and 1.6:1 has been used, so the final surface composition was found to be $Ru_{55}H_{53}$ and $Ru_{55}H_{70}$, respectively, as illustrated in Figure S15. In all carbon models, the NP is deposited in such a way that the metal-support interaction is established preferentially through an extended (111) facet. As it can be seen, however, the anchoring of the metal species strongly depends on the nature of the support. In the case of rGO, coordination occurs exclusively on a graphitic region, since there are no point vacancies or substitutional heteroatoms that enhance accommodation. Regarding NH_2–rGO, the interaction is established mainly on a pyridinic–N_3V_1 motif, that is, three pyridine rings including a single vacancy in the center. Lastly, according to P–rGO, the adsorption of the NP takes place by the interaction with the graphitic region containing the two species of phosphorus detected experimentally, in addition to two nearby carboxyl motifs. On average for the two hydrogen surface compositions, the Ru−C bonds are in the range between 2.1 and 2.5 Å, while the few Ru–N, Ru–O, and Ru−P bonds turn out to be around 2.2, 2.2, and 2.4 Å, respectively. Regardless of the catalyst, 35 of the 55 Ru atoms are accessible, so the theoretical dispersion of the metal is around 64%. Finally, concerning the nature of the hydrogen atoms, between 60 and 62% of the H atoms showed a preference for bridging adsorption sites (coordinated to two Ru atoms) for the hydrogen coverages studied in this work. In contrast, as the H/Ru ratio increased, the hollow sites (coordinated to three Ru atoms) decreased from 32 to 26% while the top ones (coordinated to a single Ru atom) increased from 6 to 14%, mainly due to the evident crowding of the metal surface for the ratio $Ru_{55}H_{70}$ with respect to $Ru_{55}H_{53}$.

Structure-property analysis of the supported $Ru_{55}H_{53}$ and $Ru_{55}H_{70}$ NPs using nearest-neighbor (NN) search revealed average Ru−Ru and Ru−H bond lengths of 2.56 and 1.75 Å, respectively, with no significant differences between the supports and stoichiometry studied in this work. It is found that the average NN coordination number (CN) of Ru−Ru and Ru−H is 7.96 and 2.15, respectively, in the case of the former with differentiated values depending on the Ru atoms located in the core (CN_{max}=12) and on the surface (CN_{min}=5) of the NP. In addition, the mean interatomic distance (MIAD) was calculated, which reflects the effective radius of a cluster, obtaining a value of 0.58 nm, which is equivalent to a diameter of approximately 1.2 nm, very close to the 1.5 ± 0.2 nm or 1.5 ± 0.3 nm measured by TEM in the case of Ru NPs grafted on the nitrogen- and phosphorus-doped rGO supports, and slightly under the observed diameter for the Ru-containing rGO supports (2.0 ± 0.8 nm). These numbers highlight the absence of major structural changes involved by the support.

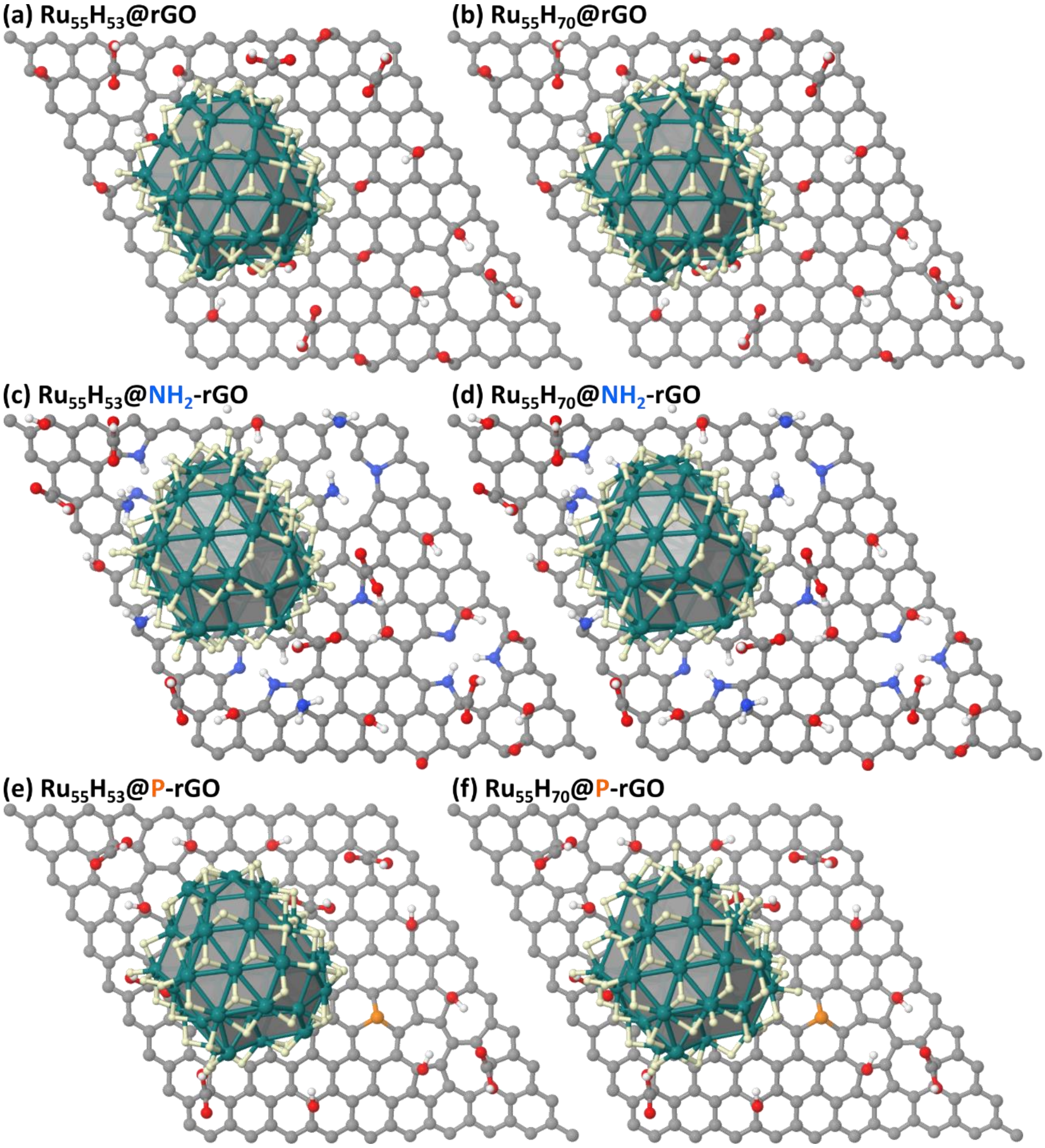

Figure S15. Top views of $Ru_{55}H_{53}$ and $Ru_{55}H_{70}$ NPs on the (**a-b**) rGO, (**c-d**) NH_2–rGO, and (**e-f**) P–rGO functionalized supports. Atomic color scheme: Ru (Celadon Green); P (Orange); O (Red); N (Blue); C (Grey); H (White); Hydrides (Yellow).

2.2. Volcano plots and DFT calculations of H adsorption energies

Volcano plots were calculated using the Yang, Saidi and coll. electrochemical model,[21,22] which is an improvement of the seminal Nørskov model.[23] Both provide valuable theoretical frameworks for understanding the kinetics and mechanisms of the HER. As such, they can be used to rationalize the behavior of electrocatalysts, but also to design and optimize new catalysts. Both rely on the hydrogen adsorption free energy descriptor, ΔG_{H*}: the plot of the logarithm of the experimental exchange current density, log j_0, as a function of the DFT-calculated ΔG_{H*} shows that optimum catalysts have a moderate adsorption strength, *i.e.* $\Delta G_{H*} \approx 0$. This is in line with the Sabatier principle.

The ΔG_{H*} descriptor is actually the dissociative adsorption energy of H_2 on the surface, *i.e.* the energy of the reaction $H_2(g) + 2* \rightarrow 2H*$ (* = active site of the surface of the electrochemical catalyst). The general formula for the average adsorption energy of n hydrides is given by: $\Delta E_{H*} = [E(nH*) - (n/2)E(H_2) - E(NP)]/n$. Given that the explicit calculation of the Gibbs free energy correction factor for all models considered in this study would be a computationally expensive task, we decided to apply a universal correction: $\Delta G_{H*} = \Delta E_{H*} + 5.5$ kcal/mol, where the 5.5 kcal/mol can be considered a representative value that accounts for the vibrational contribution to G energy for all metals.[23]

The Yang and Saidi electrochemical model for HER combines exchange current densities j_0, volcano plots, DFT adsorption energies ΔG_{H*} and Volmer-Heyrovsky pathways, according to which the overall HER proceeds through $H^+ + e^- \rightarrow ½H_2(g)$. The HER electrochemical exchange current density model is cast as a function of the rate-determining step (rds) of the Volmer, Heyrovsky and Tafel reactions:

- Volmer regime, *i.e.* rds is $H^+ + e^- \rightarrow H*$, which corresponds to $\Delta G_{H*} > 0$ of the Nørskov model

$$j_0 = ek_0C_{\text{tot}}(1-\theta)\exp\left(-\alpha\frac{\Delta G_{\text{H}*}}{k_{\text{B}}T}\right) \quad (1)$$

- Heyrovsky regime, *i.e.* rds is $H* + e^- + H^+ \rightarrow H_2$, which corresponds to $\Delta G_{H*} < 0$ of the Nørskov model

$$j_0 = ek_0C_{\text{tot}}(1-\theta)\exp\left(-(1-\alpha)\frac{\Delta G_{\text{H}*}}{k_{\text{B}}T}\right) \quad (2)$$

- the Tafel reaction is the rds at $\Delta G_{H*} = 0$

In these equations, ΔG_{H*} is the dissociative hydrogen Gibbs free energy previously introduced, α is the so-called Butler-Volmer transfer coefficient for the electrode reaction, θ is the fraction of occupied sites, C_{tot} is the total concentration of adsorbed hydrides, H*, and of protons, H^+ ($C_{\text{tot}} = C_{\text{H}^+} + C_{\text{H}*}$), and k_0 is the rate constant. Using the Langmuir model, θ can be expressed as a function of the equilibrium constant K as $\theta = K/(1+K)$, where

$$K = \exp\left(-\frac{\Delta G_{\text{H}*}}{k_{\text{B}}T}\right) \quad (3)$$

Yang and Saidi hypothesized that k_0 is metal-independent. They found $k_0 = 126$ s^{-1} by fitting the theoretical model to a database that contains j_0 and α experimental data and DFT adsorption energies, ΔG_{H*}. Volcano curves are plotted in Figure S16 as a function of α. The lower the α transfer coefficient, the smoother the slope, and the higher the exchange current density for a given ΔG_{H*} value.

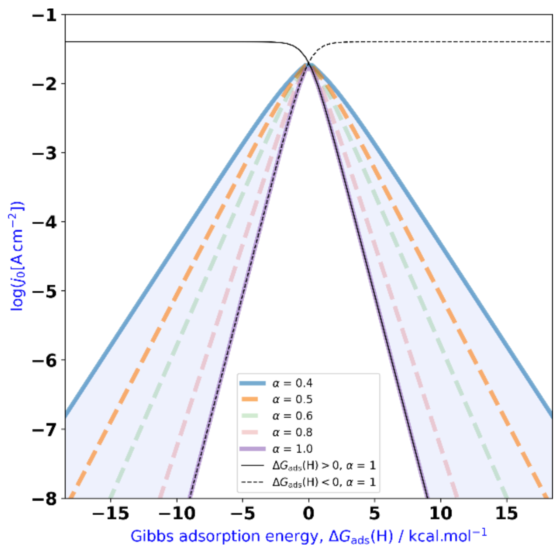

Figure S16. Volcano curves computed using eq. 1 and 2, with C_{tot} = 1 and k_0 = 126 s^{-1}.

As a consequence, the experimental knowledge of α is key to establish a quantitative correspondence between experimental exchange current densities j_0 and their theoretical counterpart, $\hat{j}_0$ (where ^ stands for "predicted"). This is clearly shown in Figure S17, where the predicted log($\hat{j}_0$) values obtained with three different models are reported as a function of the selection of experimental data of ref. 22. A linear regression has been systematically been done, as well as the calculation of the Mean Average Error (MAE) between predicted and experimental log(j_0) values. Figure S17a shows the accuracy of the seminal Nørskov model, with α fixed to 1. There is a systematic underestimation of the efficiency of the catalysts, which increases for bad catalysts. The MAE is found to be 2.806, which is quite large. There is a significant enhancement of the prediction when α is considered in the model, as shown in Figure S17b. The coefficient of determination R^2 improves (0.907 *vs*. 0.877), and the MAE decreases (1.292). There is however a significant deterioration of the model in the low log(j_0) range, *i.e*. for the least efficient catalysts. The electrochemical model used in the present work does better in this range, and is also accurate for higher log($\hat{j}_0$) values, *i.e*. to predict exchange current densities of efficient catalysts that lie close to the top of the HER volcano plot (see Figure S17c). Whilst the coefficient of determination R^2 is almost the same as that of the α-dependent Nørskov model (0.903 *vs*. 0.907), the MAE decreases (1.011).

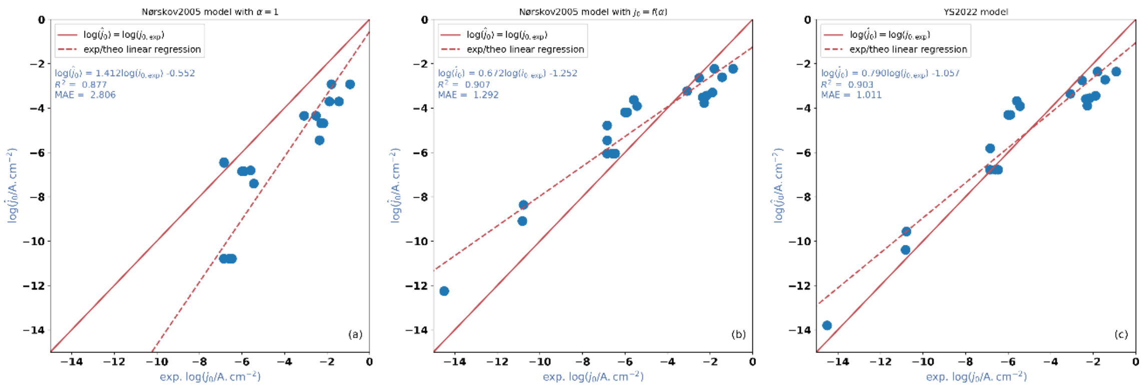

Figure S17. Predicted exchange current densities, log($\hat{j}_0$), with $\hat{j}_0$ in A·cm^{-2}, as a function of experimental data taken from the database compiled in ref. 22 (see also Table S3). (a) Seminal Nørskov model, with α = 1; (b) Nørskov model, with experimental α values; (c) Yang and Saidi model, which is expressed as a function of experimental α values. Plain claret line: hypothetical exact prediction. Dashed claret line: linear regression between log($\hat{j}_0$) and log($j_{0,\text{exp}}$); the slopes, y-intercept, R^2 and MAE are also reported on the graphs (Figs (a) and (c) are similar to the plots reported in Figure S2 of ref. 22).

	electrode	DGH/eV	logj0(YS2022)	logj0(Norskov)	logj0(Norskov,α)	logj0(exp)	exp.alpha	auth-year	doi
0	Pt/C	-0.09	-2.35	-2.920	-2.228	-1.80	0.5	J. Zheng et al. (2016)	10.1126/sciadv.1501602
1	Pt/C	-0.09	-2.35	-2.920	-2.228	-0.92	0.5	J. Durst et al. (2014)	10.1149/2.0981501jes
2	Ir	-0.14	-2.72	-3.700	-2.609	-1.44	0.5	J. Durst et al. (2014)	10.1149/2.0981501jes
3	Ir	-0.14	-3.44	-3.700	-3.296	-1.89	0.8	J. Zheng et al. (2016)	10.1126/sciadv.1501602
4	Pd	-0.18	-2.75	-4.340	-2.641	-2.52	0.4	A. Safavi et al. (2014)	10.1016/j.fuel.2013.10.016
5	Pd	-0.18	-3.35	-4.340	-3.230	-3.08	0.6	J. Zheng et al. (2016)	10.1126/sciadv.1501602
6	Rh/C	-0.20	-3.89	-4.680	-3.766	-2.28	0.7	J. Durst et al. (2014)	10.1149/2.0981501jes
7	Rh/C	-0.20	-3.55	-4.680	-3.430	-2.17	0.6	J. Zheng et al. (2016)	10.1126/sciadv.1501602
8	Ru	-0.25	-3.59	-5.450	-3.514	-2.35	0.5	A. Kuhn et al. (1970)	10.1016/S0022-0728(70)80194-2
9	Cu	0.30	-5.81	-6.469	-5.455	-6.84	0.8	N. Pentland et al. (1957)	10.1149/1.2428530
10	Cu	0.25	-5.81	-6.440	-4.779	-6.84	0.8	N. Pentland et al. (1957)	10.1149/1.2428530
11	Co	-0.37	-3.91	-7.400	-3.901	-5.44	0.4	K. Kawashima et al. (2020)	10.1021/acsaem.0c00321
12	Ni	-0.33	-3.68	-6.810	-3.632	-5.59	0.4	E. Navarro-Flores et al. (2005)	10.1016/j.molcata.2004.10.029
13	Au(111)	0.50	-6.78	-10.790	-6.046	-6.60	0.5	J. Perez et al. (1998)	10.1021/jp9831987
14	Au(111)	0.50	-6.78	-10.790	-6.046	-6.47	0.5	S. Štrbac et al. (2018)	10.1149/2.0441815jes
15	PolyAu	0.50	-6.78	-10.790	-6.046	-6.85	0.5	J. Perez et al. (1998)	10.1021/jp9831987
16	Re	-0.33	-4.30	-6.850	-4.189	-5.90	0.9	J.G. Rivera et al. (2019)	10.1016/j.ijhydene.2019.08.212
17	Re	-0.33	-4.30	-6.850	-4.189	-6.00	0.9	R. Garcia-Garcia et al. (2015)	10.1007/s12678-014-0240-z
18	Bi	1.07	-13.80	-20.830	-12.250	-14.50	0.6	Y.M. Wu et al. (2005)	10.1016/j.jpowsour.2004.10.019
19	Cd	1.03	-9.56	-19.810	-8.362	-10.77	0.4	J.O.M. Bockris et al (1964)	10.1016/0013-4686(64)80003-7
20	In	0.91	-10.39	-17.890	-9.089	-10.82	0.5	J.N. Butler et al. (1965)	10.1149/1.2423502

Table S3. Reproduction of the database used in ref. 22 to compare the predictive performance of electrochemical models with respect to experimental values (values used in the in-house Jupyter Notebook workflow developed for this study). logj0(YS2022): Yang and Saidi electrochemical model; logj0(Nørskov): seminal Nørskov model with α = 1; logj0 (Nørskov,α): seminal Nørskov model with experimental α. DGH/eV is ΔG_{H*}. The logj0 (Nørskov, α) predicted values were not reported in Table S3 of ref. 22.

The application of the electrochemical model to the rGO systems leads to the volcano curves plotted in Figure S18. If the electrochemical model is reliable, and provided that DFT adsorption energies are accurate enough and that the structural models developed in this study (*vide infra*) are close to the actual experimental systems, these plots show that in average ΔG_{H*} is expected

to be found at ≈ -1, -3 and -5 kcal·mol^{-1} for **Ru-r@P-rGO**, **Ru-r@NH$_2$-rGO** and **Ru-r@rGO**, respectively. This is obviously a real challenge given the complexity of the system and the standard accuracy of DFT, so it would be satisfying to find the same trend.

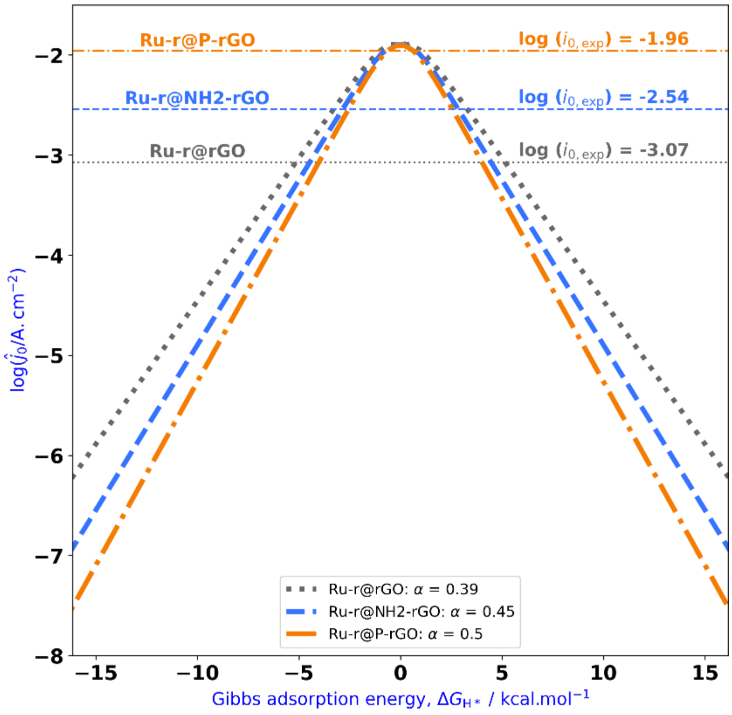

Figure S18. Volcano plots predicted after the Yang, Saidi and coll. electrochemical model,[21,22] using α transfer coefficients found in the present study (see Table 1 in the MS), and with k_0: 126 s^{-1} and C_{tot}: 1. The experimental log(j_0) values are also reported.

2.3. Evaluation of the range effect of the supports on H adsorption energies

Instead of calculating the adsorption energy of a few hydrides selected here and there on the surface, a procedure was defined to evaluate the effect of the radius of action of the support on the hydride's adsorption energy. The methodology is summarized in Figure S19a, where R is the distance between the support and the atoms of the NP. It primarily consists in discretizing the interaction distance, R, by creating a set of contiguous intervals that span their range of values between 0 and R_{max}, where the support is the origin of the R scale. The m hydrides H* that lie in a given [R_I, R_{I+1}] range are removed in order to calculate the corresponding ΔE_{H^*} value: $\Delta E_{H^*} = [E(nH^*) - E((n-m)H^*) - (m/2)E(H_2)]/m$.

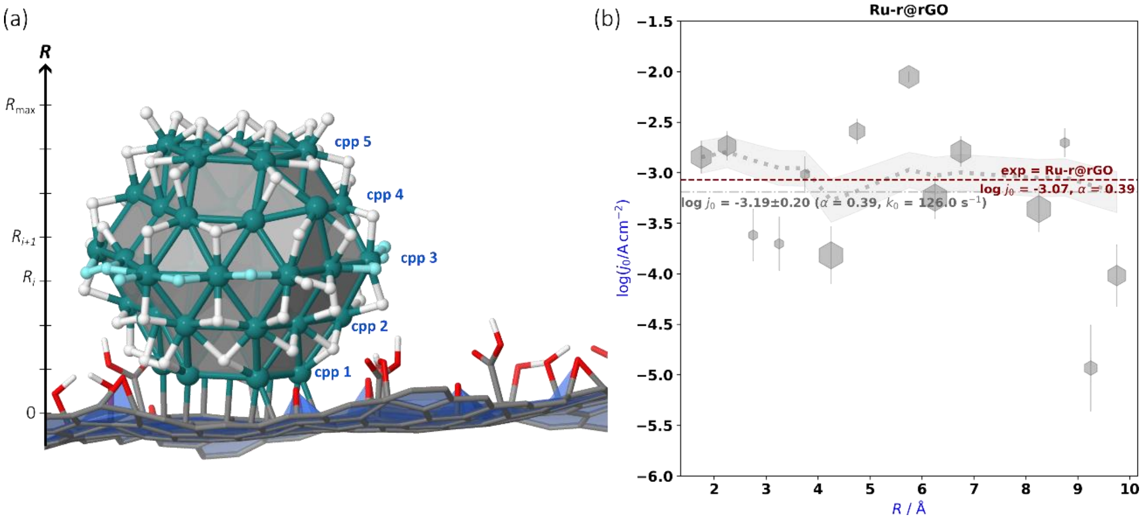

Figure S19. Range effect of the support on the H adsorption energies for the Ru-r@rGO case: (a) discretization of the distance *R* between the support and the atoms of the nanoparticle for this $Ru_{55}H_{53}$@rGO model. Cyan atoms are hydrides that lie in a [R_i, R_{i+1}] interval. The close-packed planes (cpp) are itemized from bottom to top; (b) example of log $\hat{j}_0$ plotted as a function of *R*: hexagonal markers = value calculated for a given [R_i, R_{i+1}] interval (their size is proportional to $n_H(R)$, the number of hydrides in the corresponding interval; error bars are calculated considering a possible arbitrary error of ±0.05 in the determination of the α value), thick dashed grey line = weighted average of log $\hat{j}_0$ between 0 and a given distance R_i (see text) - the area surrounding the weighted average value of log $\hat{j}_0$ takes into account any experimental determination ±0.05 error on α, horizontal dash dotted grey line = weighted average of log $\hat{j}_0$ between 0 and R_{max}, horizontal dashed claret line = experimental value.

The log $\hat{j}_0$ value is then easily calculated for each range, as described in section 2.2. It can be plotted for each interval, as shown in Figure S19b (hexagonal markers). A weighted average of log $\hat{j}_0$ can also be calculated as

$$\overline{\log\hat{j}_0(R_I)} = \frac{\sum_{R=0}^{R_I} n_{\mathrm{H}}(R)\log\hat{j}_0(R)}{\sum_{R=0}^{R_I} n_{\mathrm{H}}(R)} \quad (4)$$

which is plotted as a thick dotted grey line in Figure S19b. It is then possible to report the log $\hat{j}_0$ values calculated on all [R_i, R_{i+1}] intervals on a volcano plot defined from the previously described Yang and Saidi model, using the experimental α values. This is what is reported in Figure S20 (blue diamonds). This figure supports various analysis, as explained in the figure itself as well as in its caption. The difference between the horizontal blue and light blue dashed lines can be interpreted as the direct short-range effect of the support on the HER performance. On the

other hand, on an unadsorbed model, hydrides would be better distributed on the surface. The influence of the H---H closer interaction on $\log\hat{\imath}_0$ in $Ru_{55}H_{53}$@NH_2-rGO with respect to the free $Ru_{55}H_{53}$ model is also evaluated by considering two systems: $Ru_{55}H_{53}$ after its supported counterpart and $Ru_{55}H_{53}$ with hydrides distributed on the whole metal surface of the Ru_{55} metal part, including the bottom of the Ru_{55} model (named $Ru_{55}H_{53}$ eq. in the caption of Figure S20).

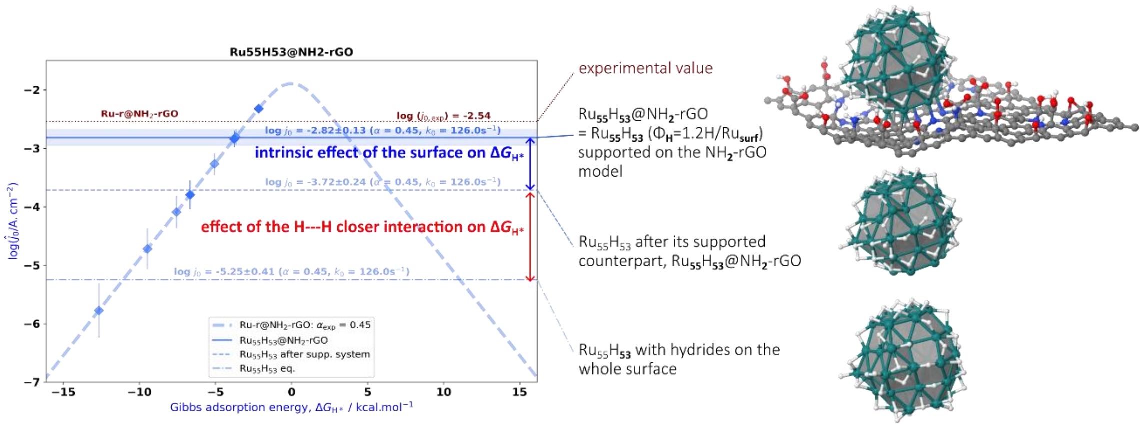

Figure S20. Volcano plot for the Ru-r@NH_2-rGO, after the Yang and Saidi model and using the experimental α value and the "universal" k_0 rate constant calculated in ref. 22. The experimental log j_0 is also shown as a horizontal dotted claret line. Blue diamonds: log $\hat{j}_0$ values calculated for the $Ru_{55}H_{53}$@NH_2-rGO model in each [R_i, R_{i+1}] interval, between R_0 = 0 and R_l = 3 Å**.** Light blue diamonds: log $\hat{j}_0$ values calculated for the same intervals, but on the unsupported $Ru_{55}H_{53}$ model. The corresponding $\overline{\log\hat{j}_0(3)}$ averaged value is also reported in both cases, as horizontal light blue plain ($Ru_{55}H_{53}$@NH_2-rGO) and dashed ($Ru_{55}H_{53}$) lines. Since this value is calculated in both cases with the experimental α value found for Ru-r@NH_2-rGO, their difference can be analyzed in terms of range effect of the support. The lower dash dotted horizontal line is plotted after the $\overline{\log\hat{j}_0(3)}$ averaged value calculated for the original $Ru_{55}H_{53}$ model developed in ref. 20. In this model, hydrides are distributed on the whole surface of the Ru_{55} model, whereas in its adsorbed counterpart some hydrides at the bottom ones were moved to favor a strong coordination between the support and the NP. The meaning of the red and blue double arrows is explained on the figure.

2.4. DOS and pCOHP analysis of the support - Ru-r interactions in $Ru_{55}H_{70}$

pCOHP focuses on the strength and nature of atom-atom or orbital-orbital interactions over the energy spectrum, whereas pDOS provides a broader context by showing how specific orbitals contribute to the electronic states across the materials. Together, they deliver a comprehensive understanding of the material electronic structure and bonding. If needed, readers can familiarize themselves with such analysis by referring to ref. 40, which offers a detailed examination of the same Ru_{55} core, stabilized with hydrides and/or carbon monoxide ligands.

The DOS and pCOHP profiles of $Ru_{55}H_{70}$@X (X = rGO, NH_2-rGO, P-rGO models) are reported in Fig. S21. At first glance, they look alike, in agreement with the global similarity of the rGO supports. Some interesting numbers however arise from these two properties:

1) While the d-band center of the surface atoms is similar across all cases (approximately -3.03 eV), the d-band center of the cpp1 layer—the layer grafted onto the surface—is significantly higher in the $Ru_{55}H_{70}$@P-rGO system (-2.84 eV *vs*. -2.97 eV). Although other factors, such as local electron density, can also play a role, this in line with its superior activity towards HER.

2) The COHP Ru-surface interaction energy is strong in all cases (17.8 eV, 15.6 eV, 15.3 eV). There is no contradiction with the numbers reported in Figure 2 of the manuscript, given that this energy clue must not be confused with a bond dissociation energy (BDE), but rather considered as an intrinsic interaction energy index, not dependent of the geometry relaxation of systems when BDEs are calculated. Interestingly, it shows that an individual Ru-P interaction is significantly stronger than a Ru-C and Ru-N index (2.7 eV *vs.* 0.8 eV and 1.7 eV). Together with atomic charges and d-band center values, all these results suggest that a variation of the number of C-Ru, C-O and C-N interactions sites could modulate the activity of the Ru-r@X systems towards HER. As also revealed by the COHP analysis, the intrinsic stability of the NP is not altered by the support, each Ru-Ru bond COHP index being close to 1 eV (i.e. *ca.* 225 eV for Ru_{55}), a value similar to index energies in the $Ru_{55}H_{70}$ model (results taken from the SI of a previous study, see ref. 20; energies are given here in eV for the sake of comparison with this previous study).

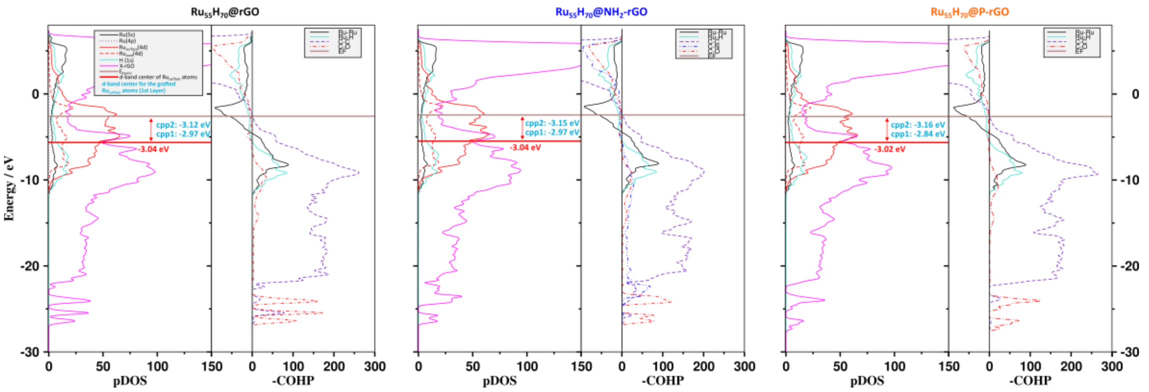

Figure S21. DOS and pCOHP analysis for the $Ru_{55}H_{70}$ Ru NP model grafted on the three supports considered in the present study (rGO, NH_2-rGO, P-rGO). The Fermi energy (brown horizontal line) and the d-band center of surface Ru atoms (red horizontal line, energy in eV) as well as the d-band center of the cpp1 and cpp2 layers of Ru atoms (in eV, cyan characters, see definition in Fig. S19) are also given. The Ru-C, Ru-N, Ru-O and Ru-P COHP profiles are not shown as they are not sufficiently intense to stand out, even when compared to the C-O interactions, which are both more numerous and stronger. A discussion is however provided in the text.

3. References

[1] Martínez-Prieto, L. M.; Puche, M.; Cerezo-Navarrete, C.; Chaudret, B. Uniform Ru Nanoparticles on N-Doped Graphene for Selective Hydrogenation of Fatty Acids to Alcohols. *J. Catal.* **2019**, *377*, 429–437. DOI: 10.1016/j.jcat.2019.07.040

[2] Mallón, L.; Cerezo-Navarrete, C.; Romero, N.; Puche, M.; García-Antón, J.; Bofill, R.; Philippot, K.; Martínez-Prieto, L. M.; Sala, X. Ru Nanoparticles Supported on Alginate-Derived Graphene as Hybrid Electrodes for the Hydrogen Evolution Reaction. *New J. Chem.* **2022**, *46* (1), 49–56. DOI: 10.1039/d1nj05215b

[3] Shervedani, R. K., Amini, A. Sulfur-doped graphene as a catalyst support: Influences of carbon black and ruthenium nanoparticles on the hydrogen evolution reaction performance. *Carbon* **2015**, *93*, 762–773. DOI: 10.1016/j.carbon.2015.05.088

[4] Chen, Z.; Lu, J.; Ai, Y.; Ji, Y.; Adschiri, T.; Wan, L. Ruthenium/Graphene-like Layered Carbon Composite as an Efficient Hydrogen Evolution Reaction Electrocatalyst. *ACS Appl. Mater. Interfaces* **2016**, *8* (51), 35132–35137. DOI: 10.1021/acsami.6b09331

[5] Liu, T.; Wang, S.; Zhang, Q.; Chen, L.; Hu, W.; Li, C. M. Ultrasmall Ru_2P Nanoparticles on Graphene: A Highly Efficient Hydrogen Evolution Reaction Electrocatalyst in Both Acidic and Alkaline Media. *Chem. Commun.* **2018**, *54* (27), 3343–3346. DOI: 10.1039/c8cc01166d

[6] Liu, T.; Feng, B.; Wu, X.; Niu, Y.; Hu, W.; Li, C. M. Ru_2P Nanoparticle Decorated P/N-Doped Carbon Nanofibers on Carbon Cloth as a Robust Hierarchical Electrocatalyst with Platinum-Comparable Activity toward Hydrogen Evolution. *ACS Appl. Energy Mater.* **2018**, *1* (7), 3143–3150. DOI: 10.1021/acsaem.8b00334

[7] Li, F.; Han, G. F.; Noh, H. J.; Ahmad, I.; Jeon, I. Y.; Baek, J. B. Mechanochemically Assisted Synthesis of a Ru Catalyst for Hydrogen Evolution with Performance Superior to Pt in Both Acidic and Alkaline Media. *Adv. Mater.* **2018**, *30* (44). DOI: 10.1002/adma.201803676

[8] Zheng, Y.; Jiao, Y.; Li, L. H.; Xing, T.; Chen, Y.; Jaroniec, M.; Qiao, S. Z. Toward Design of Synergistically Active Carbon-Based Catalysts for Electrocatalytic Hydrogen Evolution. *ACS Nano* **2014**, *8* (5), 5290–5296. DOI: 10.1021/nn501434a

[9] Qu, K.; Zheng, Y.; Zhang, X.; Davey, K.; Dai, S.; Qiao, S. Z. Promotion of Electrocatalytic Hydrogen Evolution Reaction on Nitrogen-Doped Carbon Nanosheets with Secondary Heteroatoms. *ACS Nano* **2017**, *11* (7), 7293–7300. DOI: 10.1021/acsnano.7b03290

[10] Y. H. Hung, D. Dutta, Y. J. Tseng, J. K. Chang, A. J. Bhattacharyya and C. Y. Su, *J. Phys. Chem. C*, 2019, **123**, 22202-22211. Hung, Y.-H.; Dutta, D.; Tseng, C.-J.; Chang, J.-K.; Bhattacharyya, A. J.; Su, C.-Y. Manipulation of Heteroatom Substitution on Nitrogen and Phosphorus Co-Doped Graphene As a High Active Catalyst for Hydrogen Evolution Reaction. *J. Phys. Chem. C* **2019**, *123* (36), 22202-22211. DOI: 10.1021/acs.jpcc.9b04607

[11] Li, Y.; Ai, C.; Deng, S.; Wang, Y.; Tong, X.; Wang, X.; Xia, X.; Tu, J. Nitrogen Doped Vertical Graphene As Metal-Free Electrocatalyst for Hydrogen Evolution Reaction. *Materials Research Bulletin* **2021**, *134*, 111094. DOI: 10.1016/j.materresbull.2020.111094

[12] Ye, R.; Liu, Y.; Peng, Z.; Wang, T.; Jalilov, A. S.; Yakobson, B. I.; Wei, S. H.; Tour, J. M. High Performance Electrocatalytic Reaction of Hydrogen and Oxygen on Ruthenium Nanoclusters. *ACS Appl. Mater. Interfaces* **2017**, *9* (4), 3785–3791. DOI: 10.1021/acsami.6b15725

[13] Wang, J.; Wei, Z.; Mao, S.; Li, H.; Wang, Y. Highly Uniform Ru Nanoparticles over N-Doped Carbon: pH and Temperature-Universal Hydrogen Release from Water Reduction. *Energy Environ Sci* **2018**, *11* (4), 800–806. DOI: 10.1039/c7ee03345a

[14] Shi, Y.; Dai, W.; Wang, M.; Xing, Y.; Xia, X.; Chen, W. Bioinspired Construction of Ruthenium-Decorated Nitrogen-Doped Graphene Aerogel as an Efficient Electrocatalyst for Hydrogen Evolution Reaction. *Chem Res Chin Univ* **2020**, *36* (4), 709–714. DOI: 10.1007/s40242-020-0167-2

[15] Zheng, B.; Ma, L.; Li, B.; Chen, D.; Li, X.; He, J.; Xie, J.; Robert, M.; Lau, T. C. pH Universal Ru@N-Doped Carbon Catalyst for Efficient and Fast Hydrogen Evolution. *Catal. Sci. Technol.* **2020**, *10* (13), 4405–4411. DOI: 10.1039/c9cy02552a

[16] Creus, J.; Drouet, S.; Suriñach, S.; Lecante, P.; Collière, V.; Poteau, R.; Philippot, K.; García-Antón, J.; Sala, X. Ligand-Capped Ru Nanoparticles as Efficient Electrocatalyst for the Hydrogen Evolution Reaction. *ACS Catal.* **2018**, *8* (12), 11094–11102. DOI: 10.1021/acscatal.8b03053

[17] Gerber, I. C.; Serp, P. A Theory Experience Description of Support Effects in Carbon-Supported Catalysts. *Chem. Rev.* **2020**, *120* (2), 1250–1349. DOI: 10.1021/acs.chemrev.9b00209

[18] Kotakoski, J.; Meyer, J. C.; Kurasch, S.; Santos-Cottin, D.; Kaiser, U.; Krasheninnikov, A. V. Stone-Wales-Type Transformations in Carbon Nanostructures Driven by Electron Irradiation. *Phys. Rev. B* **2011**, *83* (24), 245420. DOI: 10.1103/physrevb.83.245420

[19] Navarro-Ruiz, J.; Rivera-Cárcamo, C.; Machado, B.; Serp, P.; Del Rosal, I.; Gerber, I. C. Computational Design of Pd Nanocluster and Pd Single-Atom Catalysts Supported on O-Functionalized Graphene. *ACS Applied Nano Materials* **2021**, *4* (11), 12235–12249. DOI: 10.1021/acsanm.1c02743

[20] Cusinato, L.; Martínez-Prieto, L. M.; Chaudret, B.; Del Rosal, I.; Poteau, R. Theoretical Characterization of the Surface Composition of Ruthenium Nanoparticles in Equilibrium with Syngas. *Nanoscale* **2016**, *8* (21), 10974–10992. DOI: 10.1039/c6nr01191h

[21] Yang, T. T.; Patil, R. B.; McKone, J. R.; Saidi, W. A. Revisiting Trends in the Exchange Current for Hydrogen Evolution. *Catal. Sci. Technol.* **2021**, *11* (20), 6832–6838. DOI: 10.1039/d1cy01170g

[22] Saidi, W. A.; Yang, T. T. Reconciling the Volcano Trend with the Butler-Volmer Model for the Hydrogen Evolution Reaction. *J. Phys. Chem. Lett.* **2022**, *13* (23), 5310–5315. DOI: 10.1021/acs.jpclett.2c01411

[23] Nørskov, J. K.; Bligaard, T.; Logadottir, A.; Kitchin, J. R.; Chen, J. G.; Pandelov, S.; Stimming, U. Trends in the Exchange Current for Hydrogen Evolution. *J. Electrochem. Soc.* **2005**, *152* (3), J23–J26. DOI: 10.1149/1.1856988
